# Author Correction: Genome-wide association study identifies susceptibility loci for acute myeloid leukemia

**DOI:** 10.1038/s41467-021-27679-6

**Published:** 2022-01-04

**Authors:** Wei-Yu Lin, Sarah E. Fordham, Eric Hungate, Nicola J. Sunter, Claire Elstob, Yaobo Xu, Catherine Park, Anne Quante, Konstantin Strauch, Christian Gieger, Andrew Skol, Thahira Rahman, Lara Sucheston-Campbell, Junke Wang, Theresa Hahn, Alyssa I. Clay-Gilmour, Gail L. Jones, Helen J. Marr, Graham H. Jackson, Tobias Menne, Mathew Collin, Adam Ivey, Robert K. Hills, Alan K. Burnett, Nigel H. Russell, Jude Fitzgibbon, Richard A. Larson, Michelle M. Le Beau, Wendy Stock, Olaf Heidenreich, Abrar Alharbi, David J. Allsup, Richard S. Houlston, Jean Norden, Anne M. Dickinson, Elisabeth Douglas, Clare Lendrem, Ann K. Daly, Louise Palm, Kim Piechocki, Sally Jeffries, Martin Bornhäuser, Christoph Röllig, Heidi Altmann, Leo Ruhnke, Desiree Kunadt, Lisa Wagenführ, Heather J. Cordell, Rebecca Darlay, Mette K. Andersen, Maria C. Fontana, Giovanni Martinelli, Giovanni Marconi, Miguel A. Sanz, José Cervera, Inés Gómez-Seguí, Thomas Cluzeau, Chimène Moreilhon, Sophie Raynaud, Heinz Sill, Maria Teresa Voso, Francesco Lo-Coco, Hervé Dombret, Meyling Cheok, Claude Preudhomme, Rosemary E. Gale, David Linch, Julia Gaal-Wesinger, Andras Masszi, Daniel Nowak, Wolf-Karsten Hofmann, Amanda Gilkes, Kimmo Porkka, Jelena D. Milosevic Feenstra, Robert Kralovics, David Grimwade, Manja Meggendorfer, Torsten Haferlach, Szilvia Krizsán, Csaba Bödör, Friedrich Stölzel, Kenan Onel, James M. Allan

**Affiliations:** 1 Translational and Clinical Research Institute, Newcastle University Centre for Cancer, Faculty of Medical Sciences, Newcastle University, Newcastle upon Tyne, UK; 2 Section of Pediatric Hematology and Oncology, University of Chicago, Chicago, IL USA; 3 Helmholtz Zentrum München, German Research Center for Environmental Health, Neuherberg, Germany; 4 Ludwig-Maximilians-Universität München, Chair of Genetic Epidemiology, IBE, Faculty of Medicine, Munich, Germany; 5 College of Pharmacy, The Ohio State University, Columbus, OH USA; 6 Department of Medicine, Roswell Park Cancer Institute, Buffalo, NY USA; 7 Arnold School of Public Health, Department of Epidemiology & Biostatistics, University of South Carolina, Greenville, SC USA; 8 Department of Haematology, Freeman Hospital, Newcastle upon Tyne Hospitals National Health Service Foundation Trust, Newcastle upon Tyne, UK; 9 Department of Medical and Molecular Genetics, King’s College Medical School, London, UK; 10 Nuffield Department of Population Health, University of Oxford, Oxford, UK; 11 Paul O’Gorman Leukaemia Research Centre, University of Glasgow, Glasgow, UK; 12 Department of Haematology, Nottingham University Hospitals NHS Trust, Nottingham, UK; 13 Barts Cancer Institute, Queen Mary University of London, London, UK; 14 Centre for Atherothrombosis and Metabolic Disease, Hull York Medical School, Hull, UK; 15 Division of Genetics and Epidemiology, The Institute of Cancer Research, London, UK; 16 Translational and Clinical Research Institute, Faculty of Medical Sciences, Newcastle University, Newcastle upon Tyne, UK; 17 West Midlands Regional Genetics Laboratory, Birmingham Women’s Hospital, Birmingham, UK; 18 Department of Haematological Medicine, The Rayne Institute, King’s College London, London, UK; 19 National Center for Tumor Diseases NCT, Partner site Dresden, Dresden, Germany; 20 Medizinische Klinik und Poliklinik I, University Hospital Carl Gustav Carus Dresden, Technical University of Dresden, Dresden, Germany; 21 Population Health Sciences Institute, Newcastle University, Newcastle upon Tyne, UK; 22 Department of Clinical Genetics, University Hospital Rigshospitalet, Copenhagen, Denmark; 23 Institute of Hematology “L. and A. Seràgnoli”, University of Bologna, Bologna, Italy; 24 IRCCS Istituto Romagnolo per lo Studio dei Tumori (IRST) “Dino Amadori”, Meldola, Italy; 25 Hematology Service, Hospital Universitario y Politécnico La Fe, Valencia, Spain; 26 CIBERONC, Instituto de Salud Carlos III, Madrid, Spain; 27 Hematology department, Cote d’Azur University, CHU of Nice, Nice, France; 28 Division of Hematology, Medical University of Graz, Graz, Austria; 29 Università di Roma Tor Vergata, Dipartimento di Biomedicina e Prevenzione, Rome, Italy; 30 Hôpital Saint-Louis, Institut Universitaire d’Hématologie, Université Paris Diderot, Paris, France; 31 Univ. Lille, Inserm, CHU Lille, UMR-S 1172 - JPArc - Centre de Recherche Jean-Pierre AUBERT Neurosciences et Cancer, F-59000 Lille, France; 32 Department of Haematology, University College London Cancer Institute, London, UK; 33 1st Department of Internal Medicine, Semmewleis University, Budapest, Hungary; 34 3rd Department of Internal Medicine, Semmewleis University, Budapest, Hungary; 35 Department of Hematology and Oncology, Medical Faculty Mannheim, Heidelberg University, Mannheim, Germany; 36 Department of Haematology, University of Cardiff, Cardiff, UK; 37 Helsinki University Hospital Comprehensive Cancer Center, Hematology Research Unit Helsinki, University of Helsinki, Helsinki, Finland; 38 Department of Laboratory Medicine, Medical University of Vienna, Vienna, Austria; 39 MLL Munich Leukemia Laboratory, Munich, Germany; 40 HCEMM-SE Molecular Oncohematology Research Group, 1st Department of Pathology and Experimental Cancer Research, Semmelweis University, Budapest, Hungary; 41 Department of Genetics and Genomic Sciences, Icahn School of Medicine at Mount Sinai, New York, NY USA

**Keywords:** Acute myeloid leukaemia, Cancer genomics, Risk factors

Correction to: *Nature Communications* 10.1038/s41467-021-26551-x, published online 29 October 2021.

In this article the author name Giovanni Marconi was incorrectly written as Giovani Marconi. The original article has been corrected.

