# Supplementary material for: Genome-wide association study identifies susceptibility loci for acute myeloid leukemia
Source: Nat Commun. 2021 Oct 29;12:6233. doi: 10.1038/s41467-021-26551-x (PMC8556284; doi:10.1038/s41467-021-26551-x)
Supplement: Supplementary file 1 — Supplementary Information [file 41467_2021_26551_MOESM1_ESM.pdf]

Genome-wide association study identifies susceptibility loci for acute myeloid leukemia

Lin et al

Supplementary Figures

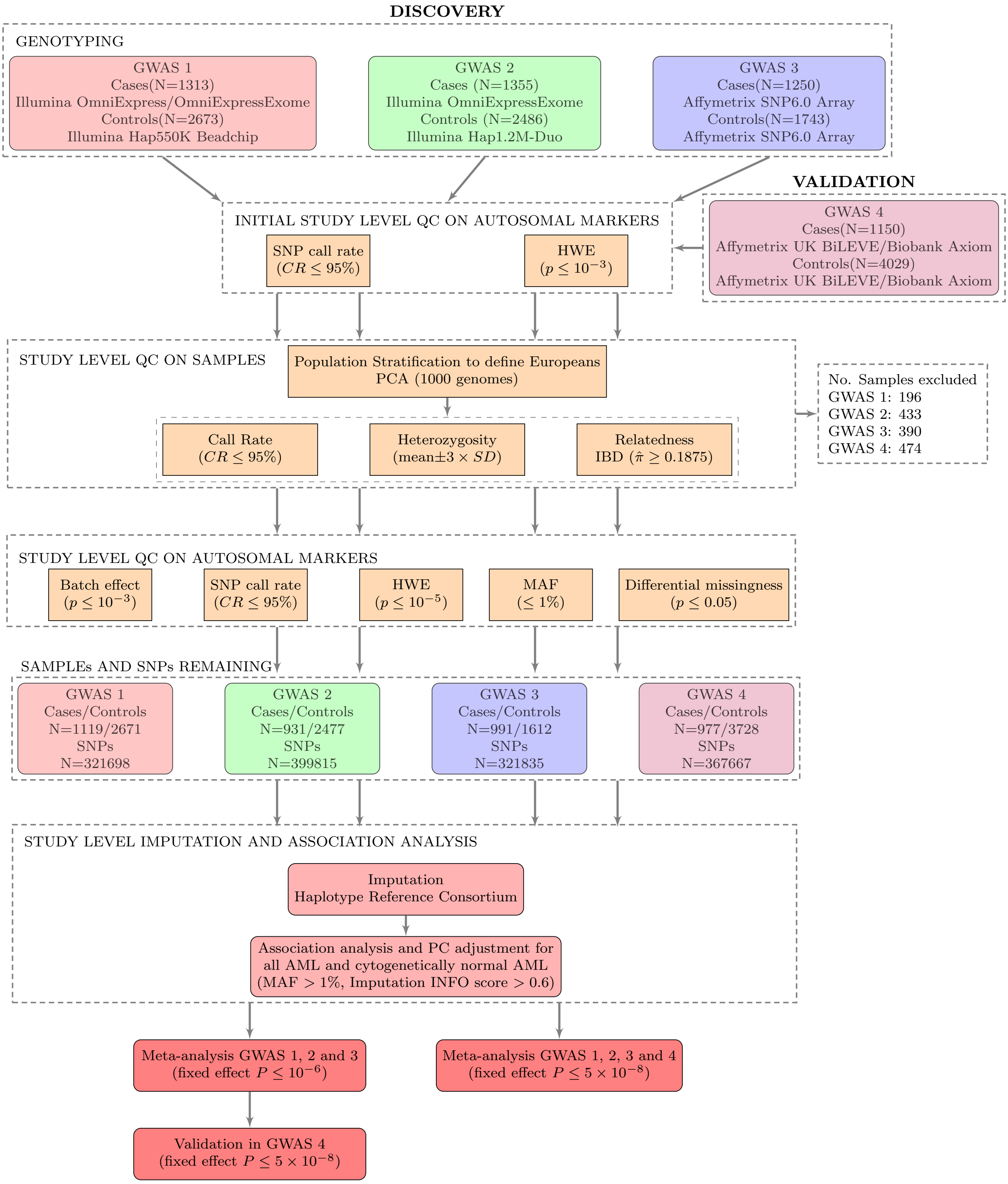

Supplementary Figure 1: **Details of data analysis workflow and quality control filters applied to each AML GWAS.** SNPs with a call rate $< 98\%$ or showing significant deviation from Hardy-Weinberg ($P \leq 10^{-3}$) were excluded. SNPs that showed significant differences ($P < 10^{-3}$) between genotype batches and with significant differences ($P < 0.05$) in missingness between cases and controls were also excluded. Samples were excluded due to low call rate ($< 95\%$), ancestry (principal components analysis), relatedness ($\pi \geq 0.1875$) or heterozygosity (mean $\pm 3 \times$SD). Imputed SNPs with information score < 0.6 or MAF < 0.01 were excluded.

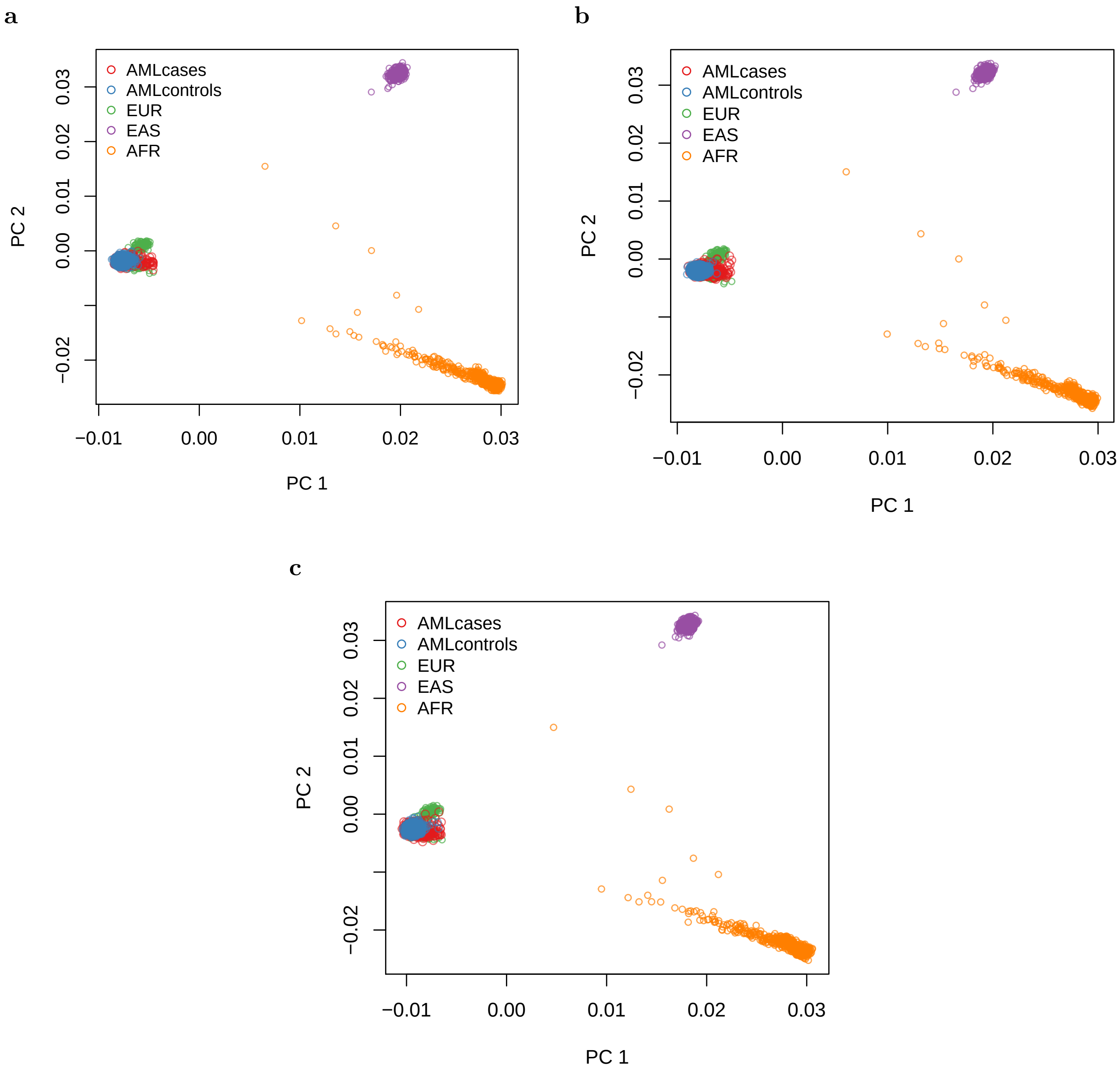

Supplementary Figure 2: **Principal component analysis (PCA) plots of ethnicity structure in a GWAS 1 b GWAS 2 c GWAS 3.** The first two principal components are shown here. European (EUR), East Asian (EAS) and African (AFR) individuals from 1000 genomes project are plotted in green, purple and orange, respectively. AML cases are plotted in red and controls are plotted in blue. PC1, principal component 1; PC2, principal component 2.

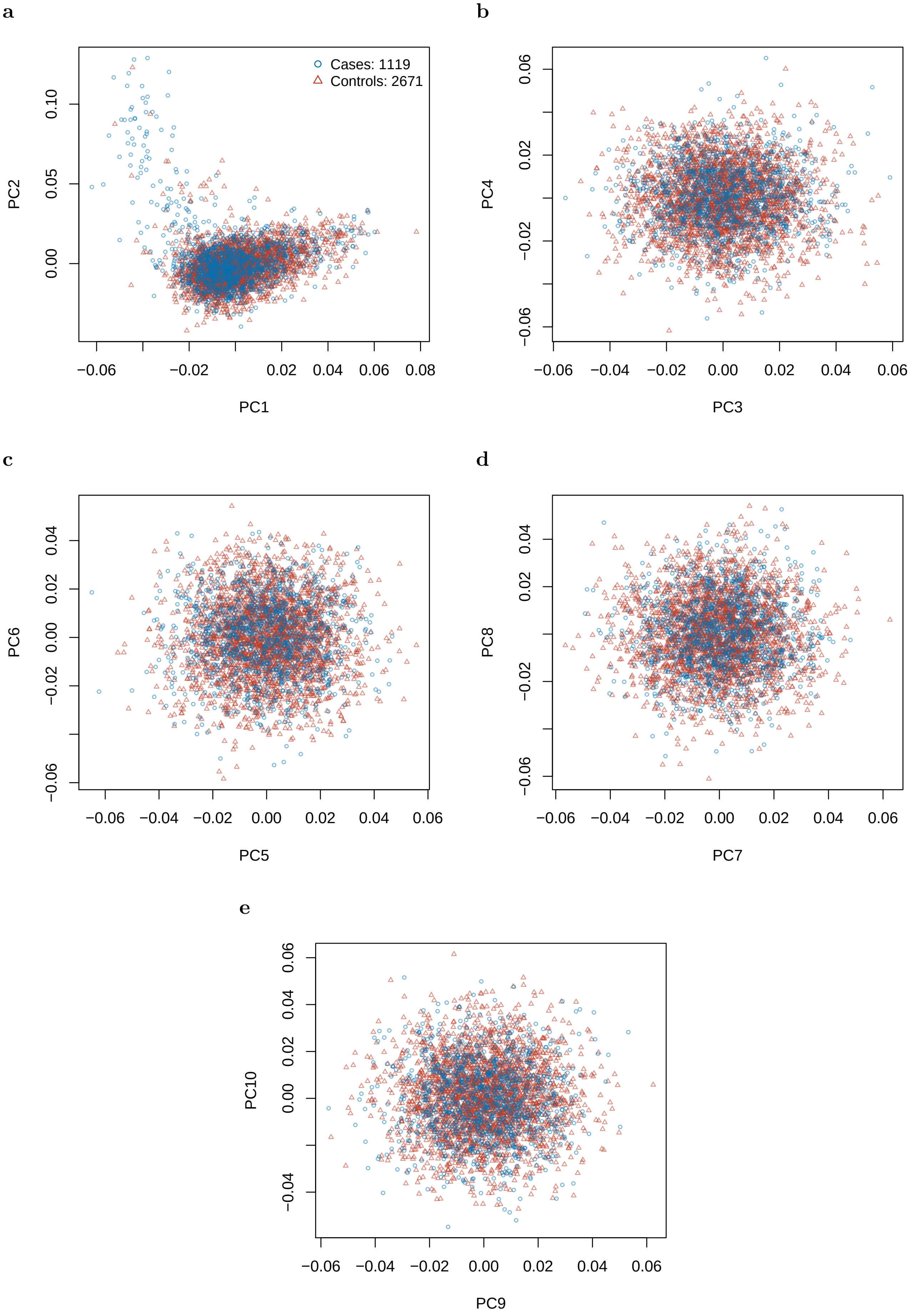

Supplementary Figure 3: PCA in GWAS 1. a PC1 and PC2. b PC3 and PC4. c PC5 and PC6. d PC7 and PC8. e PC9 and PC10.

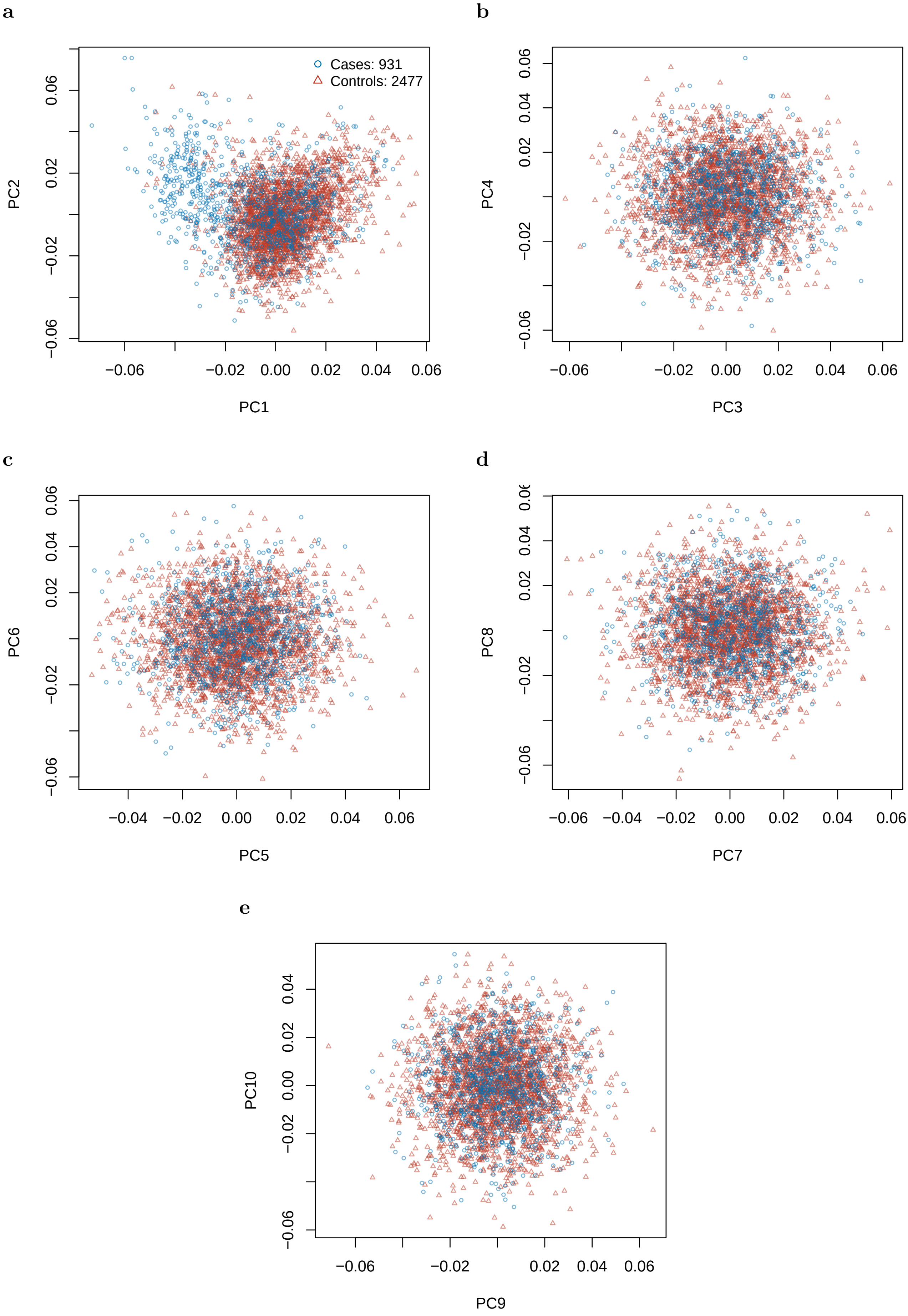

Supplementary Figure 4: PCA in GWAS 2. a PC1 and PC2. b PC3 and PC4. c PC5 and PC6. d PC7 and PC8. e PC9 and PC10.

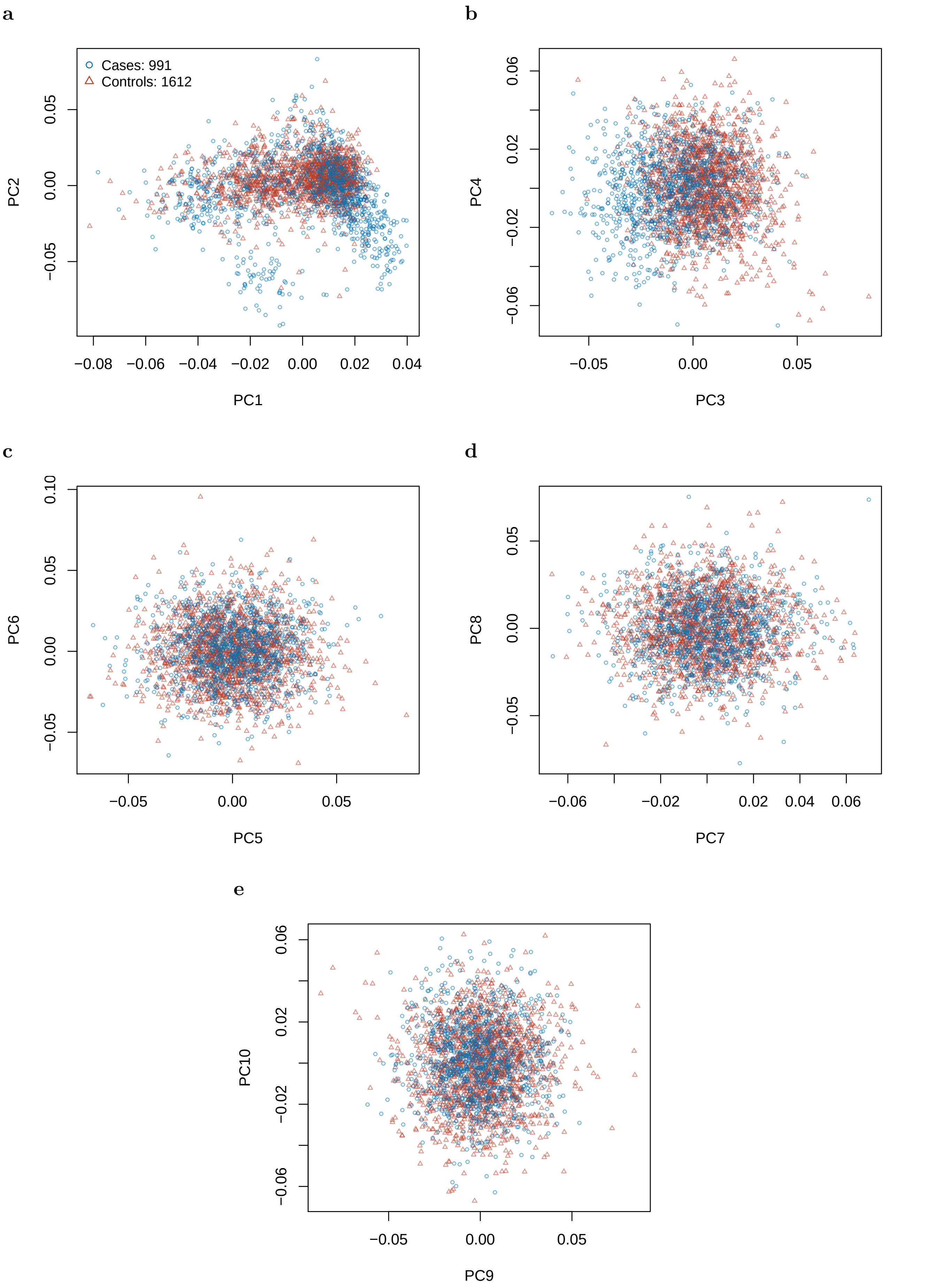

Supplementary Figure 5: PCA in GWAS 3. a PC1 and PC2. b PC3 and PC4. c PC5 and PC6. d PC7 and PC8. e PC9 and PC10.

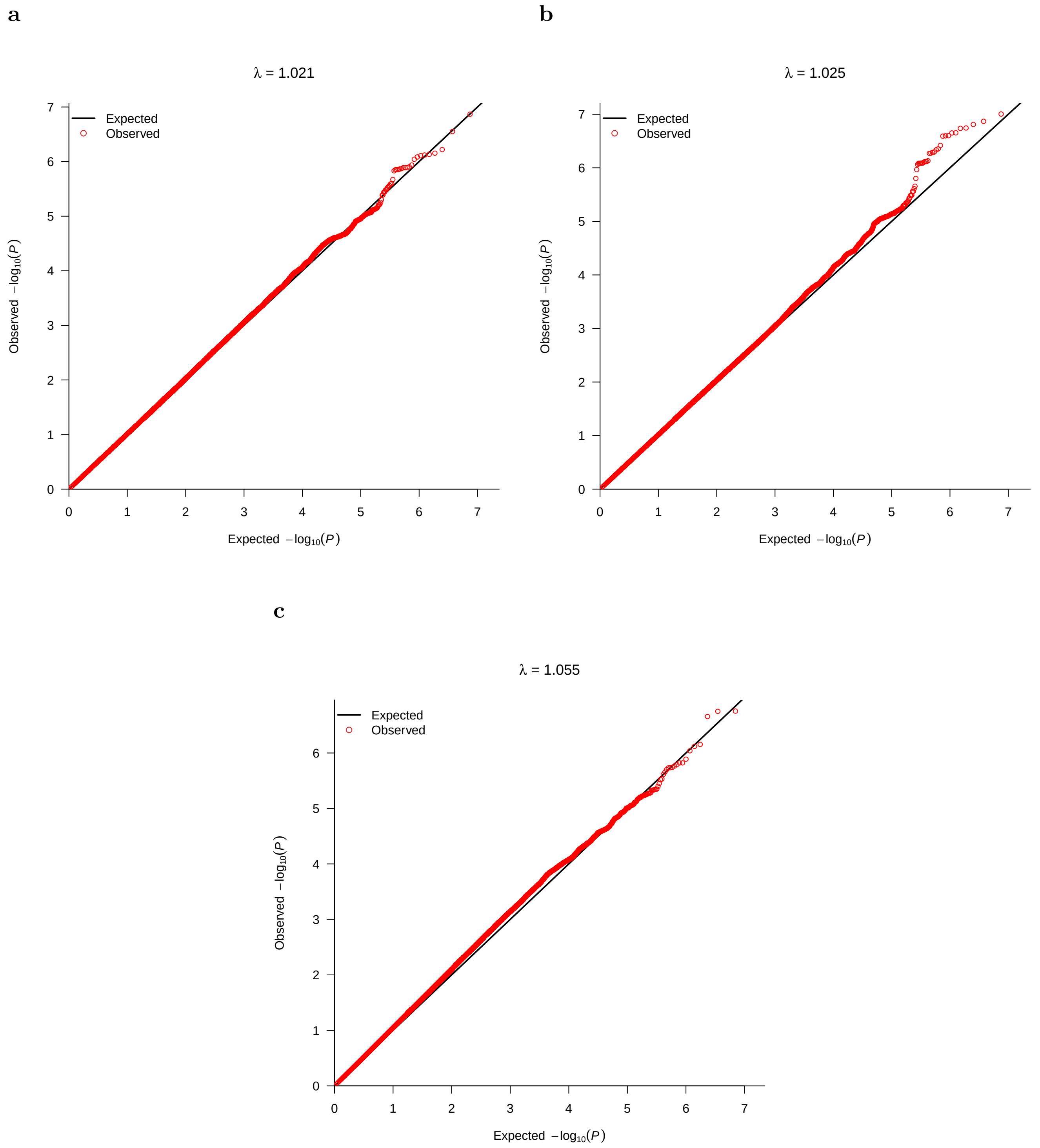

Supplementary Figure 6: **Quantile-Quantile plots of observed P values versus expected P values from association results for all AML in a GWAS 1 b GWAS 2 c GWAS 3.** For each GWAS, association tests were performed for all AML cases assuming an additive genetic model, with nominally significant principal components included in the analysis as covariates. Association P values (observed versus expected) on imputed genotype data (MAF > 0.01, INFO > 0.6) are plotted for all AML cases. All statistical tests were two-sided and no adjustments were made for multiple comparisons.

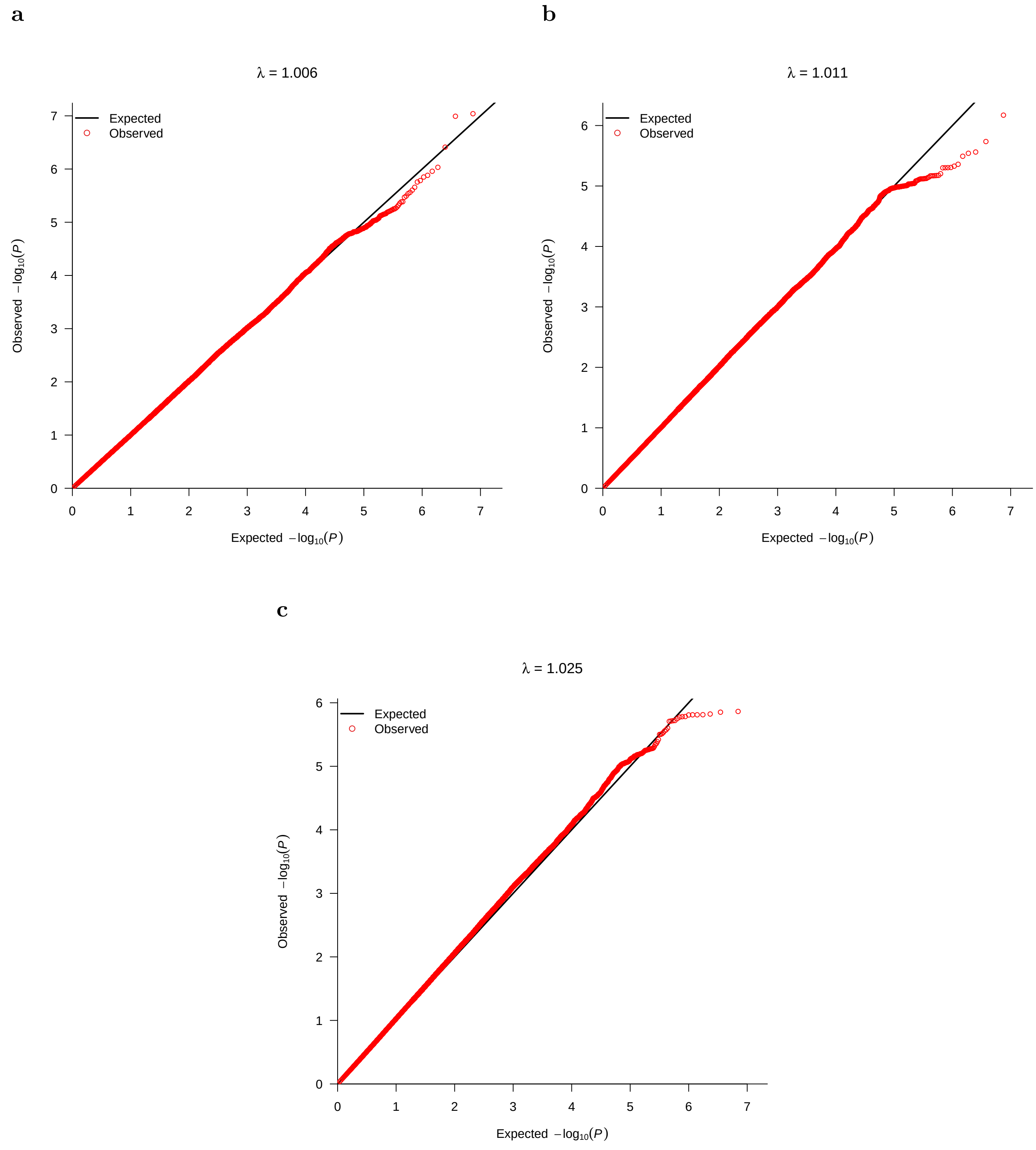

Supplementary Figure 7: **Quantile-Quantile plots of observed P values versus expected P values from association results for cytogenetically normal AML in a GWAS 1 b GWAS 2 c GWAS 3.** For each GWAS, association tests were performed for cytogenetically normal AML cases assuming an additive genetic model, with nominally significant principal components included in the analysis as covariates. Association P values (observed versus expected) on imputed genotype data (MAF > 0.01, INFO > 0.6) are plotted for cytogenetically normal AML cases. All statistical tests were two-sided and no adjustments were made for multiple comparisons.

a

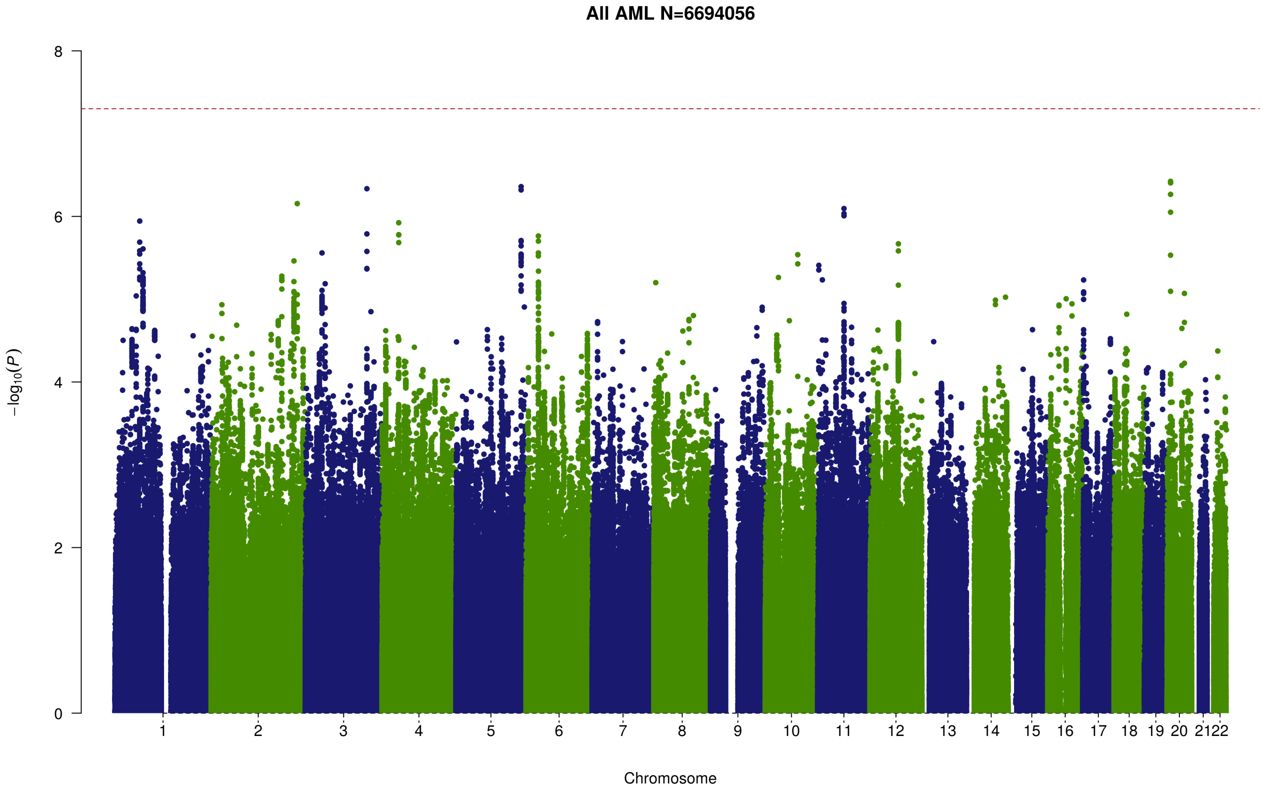

b

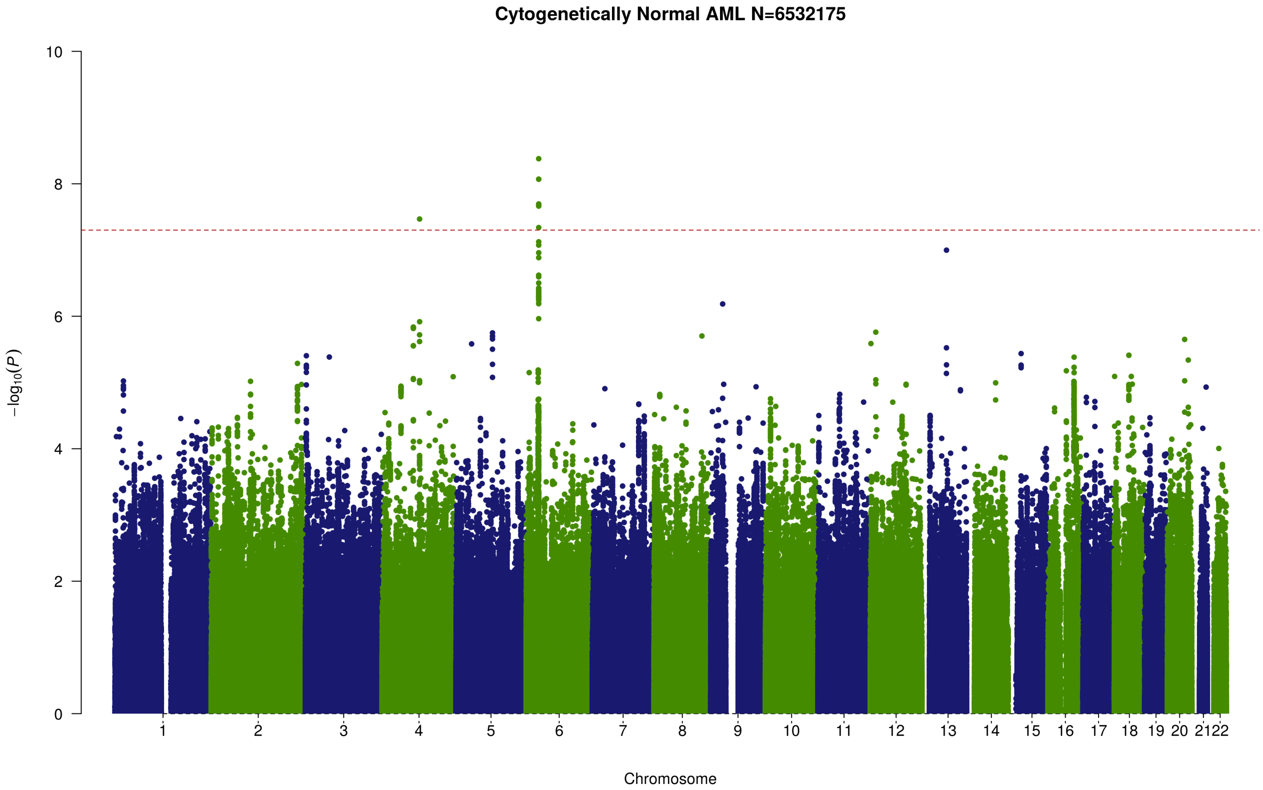

Supplementary Figure 8: **Manhattan plot from meta-analysis of 3 genome-wide association studies for all AML a and cytogenetically normal AML b.** For each GWAS, association tests were performed for all AML cases and cytogenetically normal AML assuming an additive genetic model, with nominally significant principal components included in the analysis as covariates. Association summary statistics were combined for variants common to GWAS 1, GWAS 2 and GWAS 3, in fixed effects models using PLINK. Manhattan plots show negative log_{10} (fixed effects meta P values, Y-axis) over 22 autosomal chromosomes. All statistical tests were two-sided and no adjustments were made for multiple comparisons. Horizontal red line denotes the threshold for statistical significance in a genome-wide association study ($P < 5.0 \times 10^{-8}$).

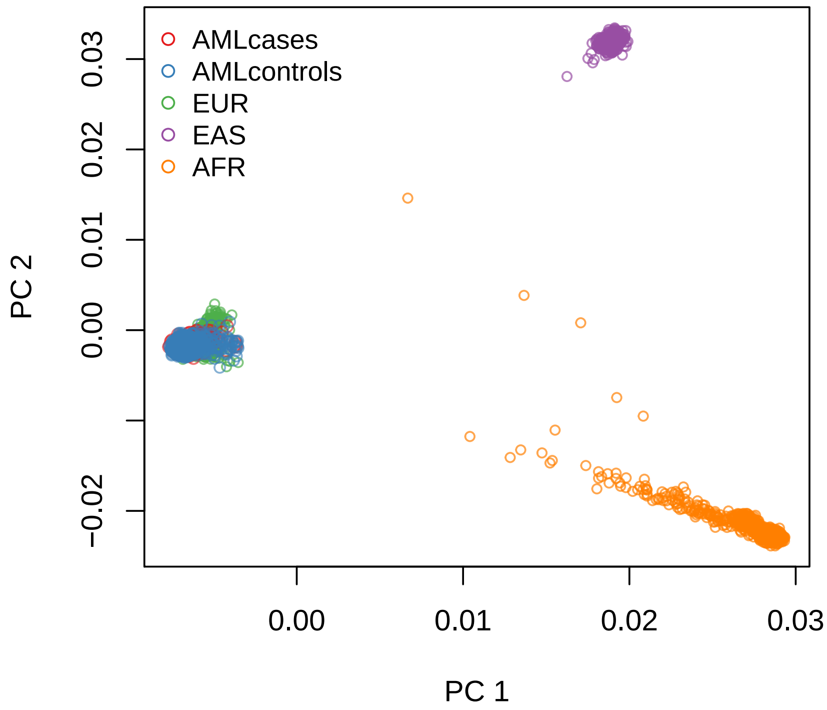

Supplementary Figure 9: **Principal component analysis (PCA) plots of ethnicity structure in GWAS 4.** The first two principal components are shown here. European (EUR), East Asian (EAS) and African (AFR) individuals from 1000 genomes project are plotted in green, purple and orange, respectively. AML cases are plotted in red and controls are plotted in blue. PC1, principal component 1; PC2, principal component 2.

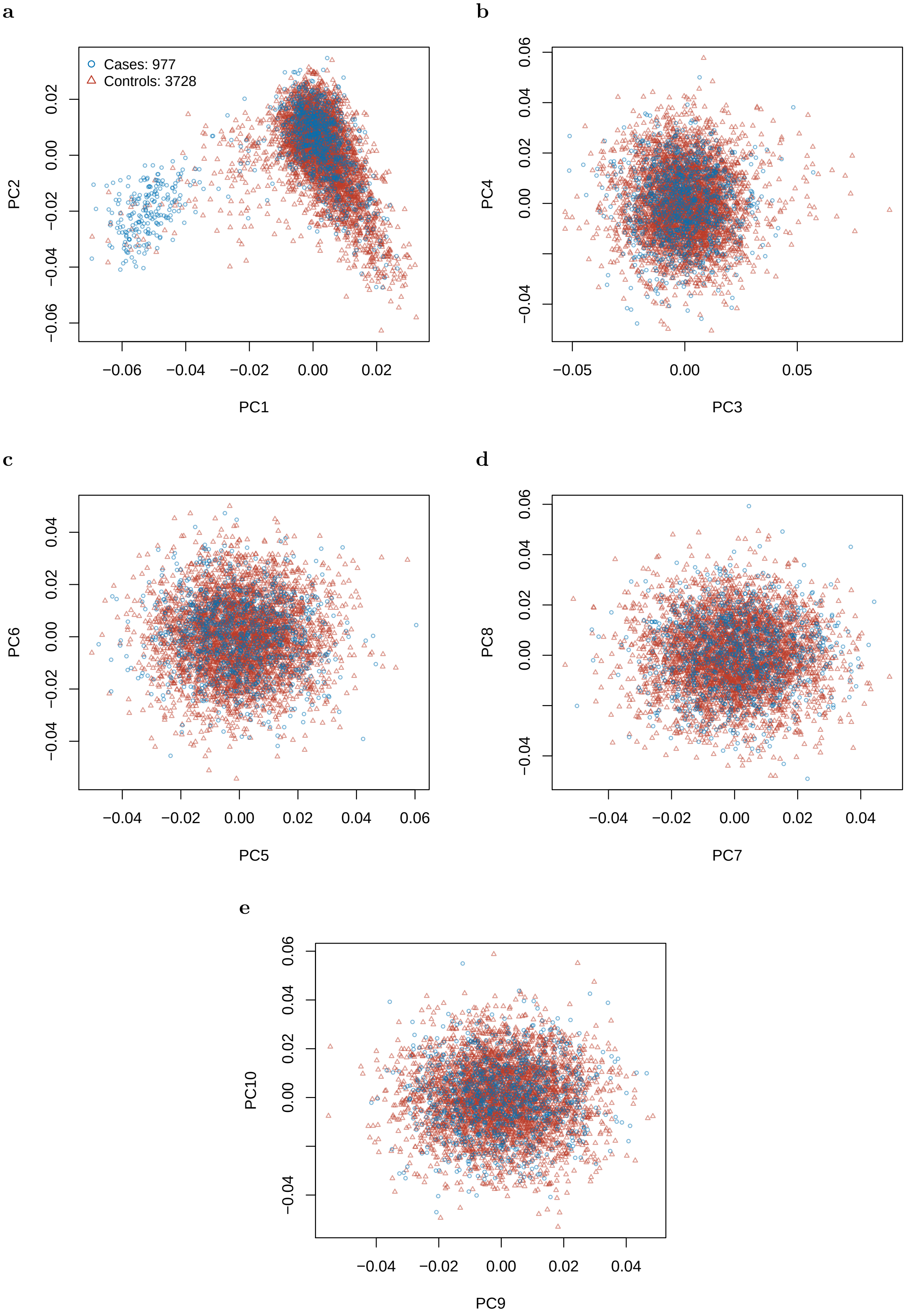

Supplementary Figure 10: PCA in GWAS 4. a PC1 and PC2. b PC3 and PC4. c PC5 and PC6. d PC7 and PC8. e PC9 and PC10.

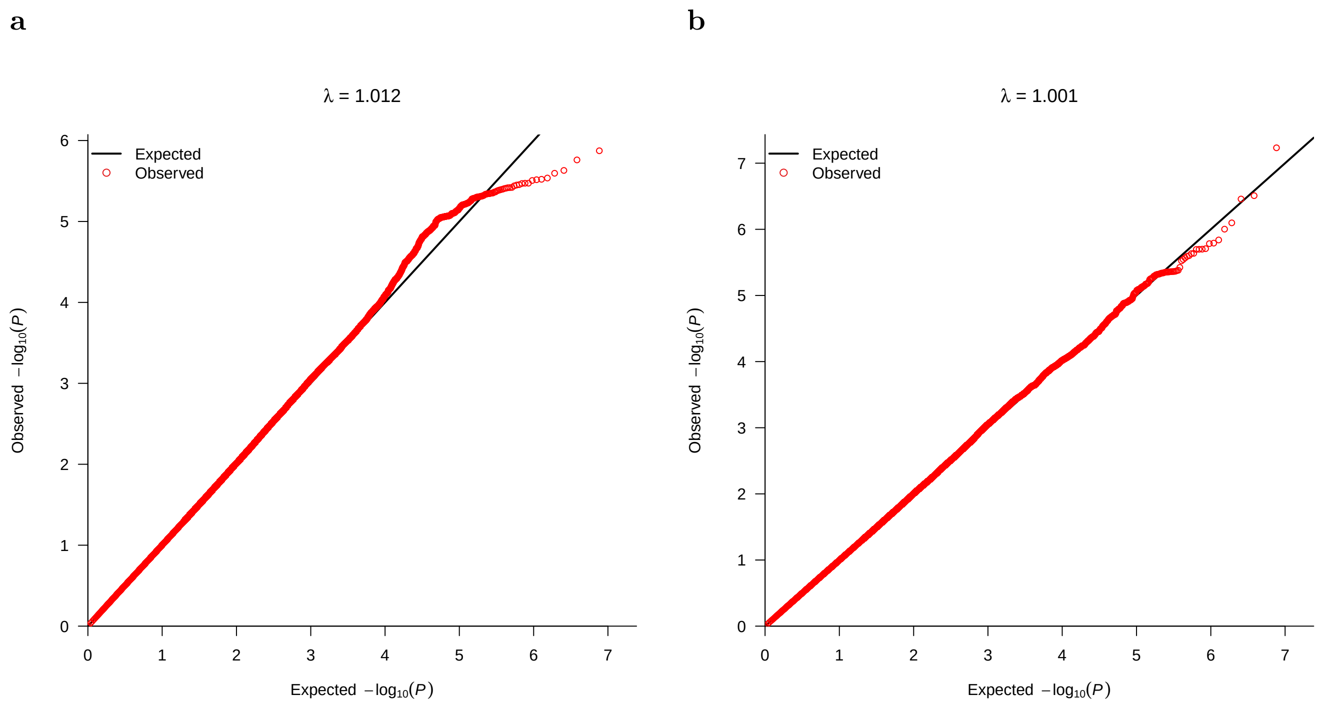

Supplementary Figure 11: **Quantile-Quantile plots of observed P values versus expected P values from association results for all AML a and cytogenetically normal AML b in GWAS 4.** For each GWAS, association tests were performed for all AML cases assuming an additive genetic model, with nominally significant principal components included in the analysis as covariates. Association P values (observed versus expected) on imputed genotype data (MAF > 0.01, INFO > 0.6) are plotted. All statistical tests were two-sided and no adjustments were made for multiple comparisons.

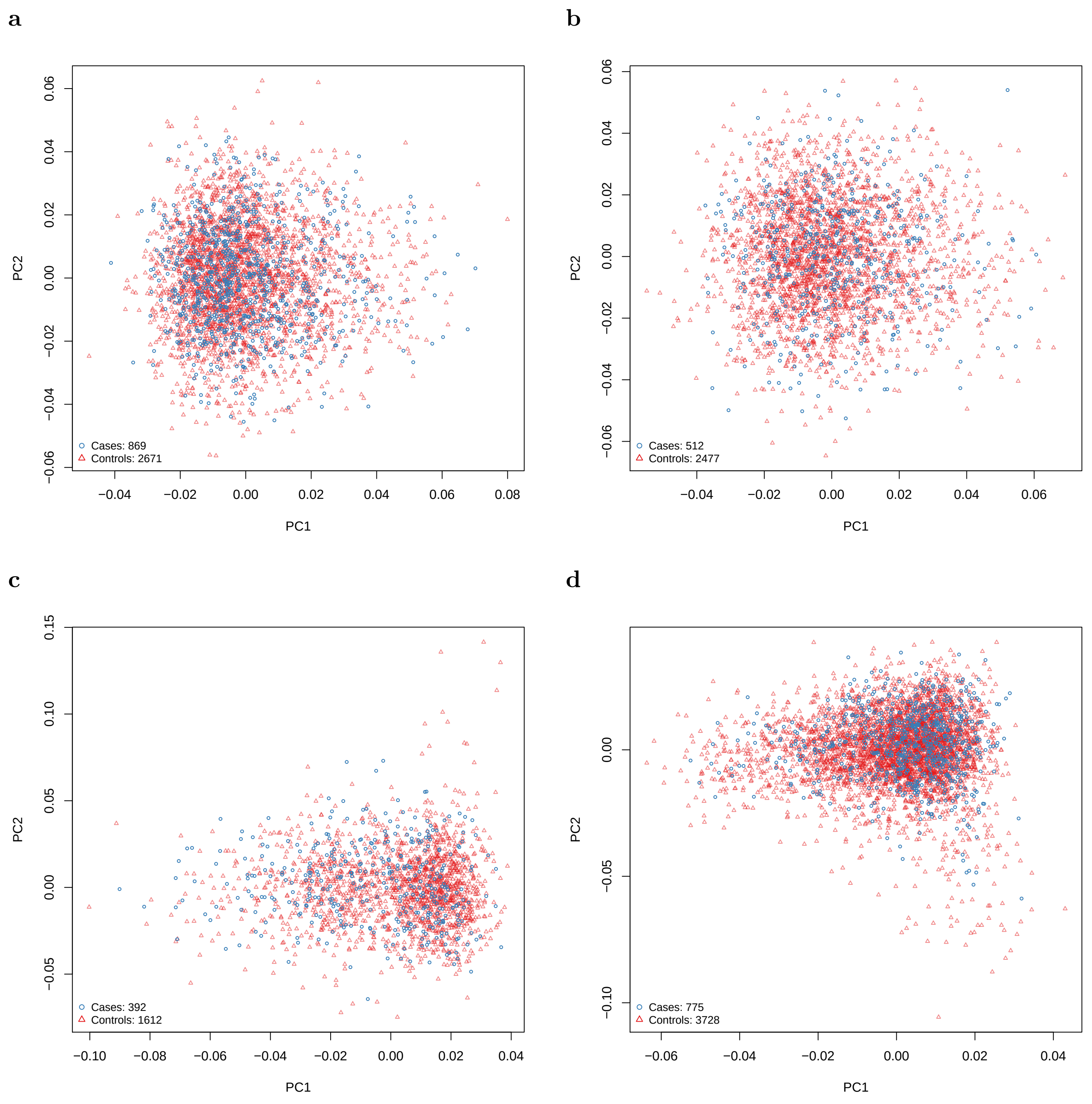

Supplementary Figure 12: **PCA showing PC1 and PC2 restricted to CEU cases and controls of UK origin for a GWAS 1, b GWAS 2 and d GWAS 4 and restricted to CEU cases of German origin for c GWAS 3.**

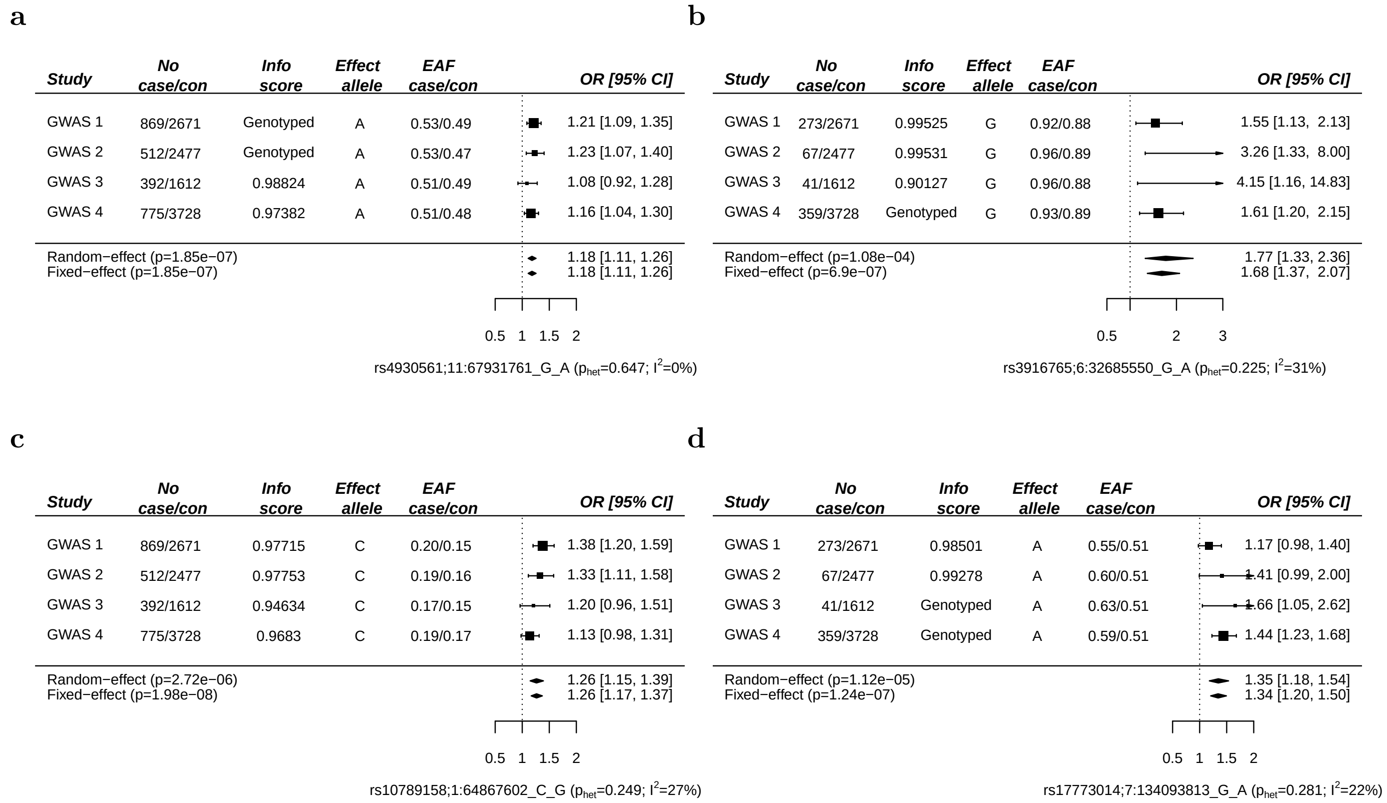

Supplementary Figure 13: **Forest plots for 4 new loci associated with acute myeloid leukemia restricted to CEU cases of UK (GWAS 1, 2 and 4) or German (GWAS 3) origin.** Study cohorts, sample sizes (case and controls (con)), imputation (info) score, effect allele, effect allele frequencies (EAF) and estimated odds ratios (OR) for rs4930561 a, rs3916765 b, rs10789158 c and rs17773014 d. The vertical line corresponds to the null hypothesis (OR=1). The horizontal lines and square brackets indicate 95% confidence intervals (95% CI). Areas of the boxes are proportional to the weight of the study. Diamonds represent combined estimates for fixed-effect and random-effect analysis. Cochrans Q statistic was used to test for heterogeneity such that $P_{HET} > 0.05$ indicates the presence of non-significant heterogeneity. The heterogeneity index, I^2 (0-100) was also measured which quantifies the proportion of the total variation due to heterogeneity. All statistical tests were two-sided and no adjustments were made for multiple comparisons.

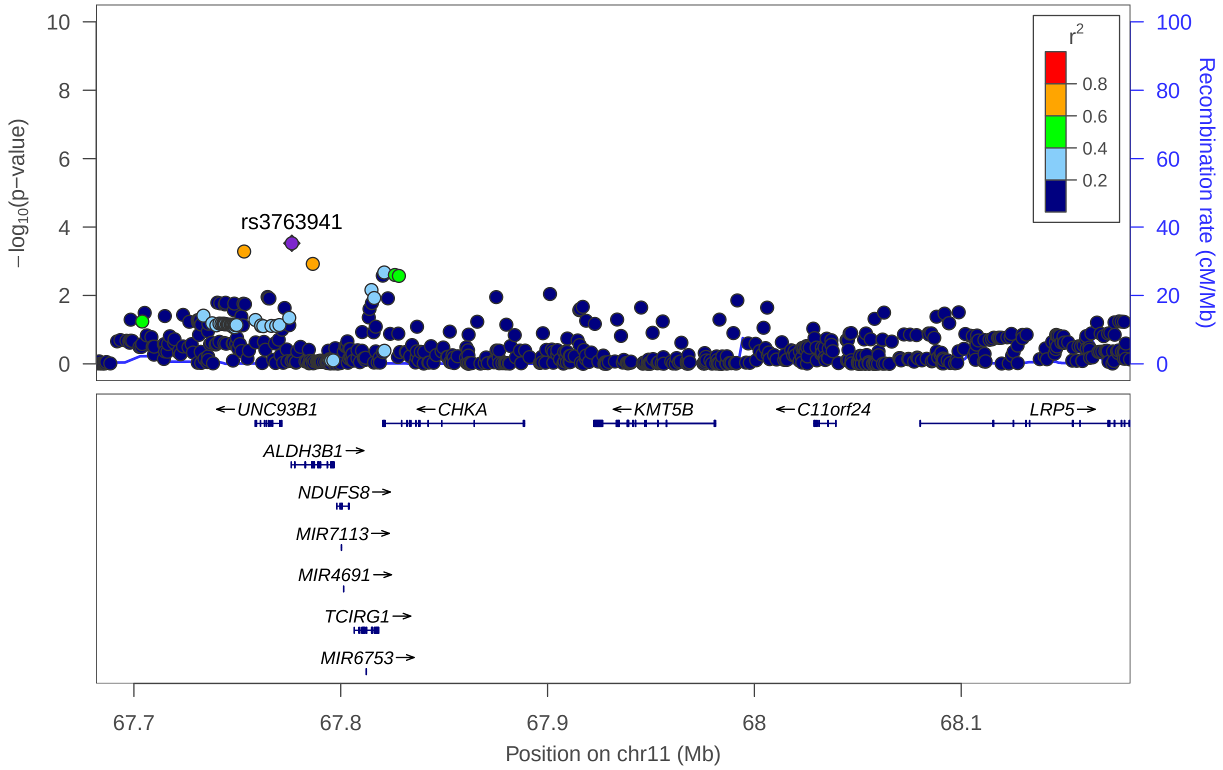

Supplementary Figure 14: **Regional association and linkage disequilibrium plots for association analysis conditioning on the top variant at the chromosome 11q13 susceptibility locus for AML.** Regional association plot showing the chromosome 11q13 AML susceptibility locus conditioning on rs4930561. For each GWAS, association tests were performed for all AML cases assuming an additive genetic model, with nominally significant principal components included in the analysis as covariates. Association summary statistics were combined for variants common to all four GWAS, in fixed effects models using PLINK. SNP coordinates based on genomic build b37/h19 are shown on the x-axis and $-log_{10}$ (P values) on the y-axis. SNPs are coloured according to their linkage disequilibrium (pairwise r^2) with the lead SNP (annotated) based on the 1000 Genomes European panel. Reference genes in the region are shown in the lower panel, with arrows indicating transcript direction, dense blocks representing exons and horizontal lines representing introns. All statistical tests were two-sided and no adjustments were made for multiple comparisons.

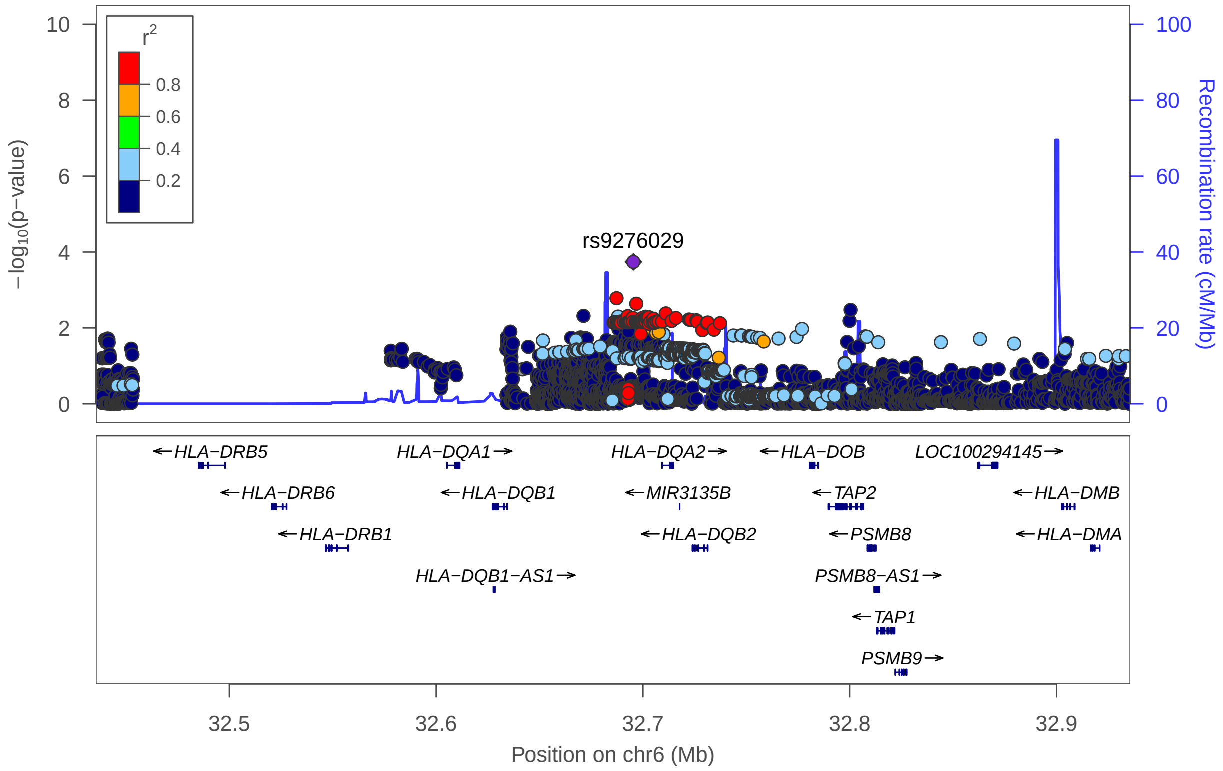

Supplementary Figure 15: **Regional association and linkage disequilibrium plots for association analysis conditioning on the top variant at the chromosome 6p21.32 susceptibility locus for cytogenetically normal AML.** Regional association plot showing the chromosome 6p21.32 AML susceptibility locus conditioning on rs3916765. For each GWAS, association tests were performed for all AML cases assuming an additive genetic model, with nominally significant principal components included in the analysis as covariates. Association summary statistics were combined for variants common to all four GWAS, in fixed effects models using PLINK. SNP coordinates based on genomic build b37/h19 are shown on the x-axis and $-log_{10}$ (P values) on the y-axis. SNPs are coloured according to their linkage disequilibrium (pairwise r^2) with the lead SNP (annotated) based on the 1000 Genomes European panel. Reference genes in the region are shown in the lower panel, with arrows indicating transcript direction, dense blocks representing exons and horizontal lines representing introns. All statistical tests were two-sided and no adjustments were made for multiple comparisons.

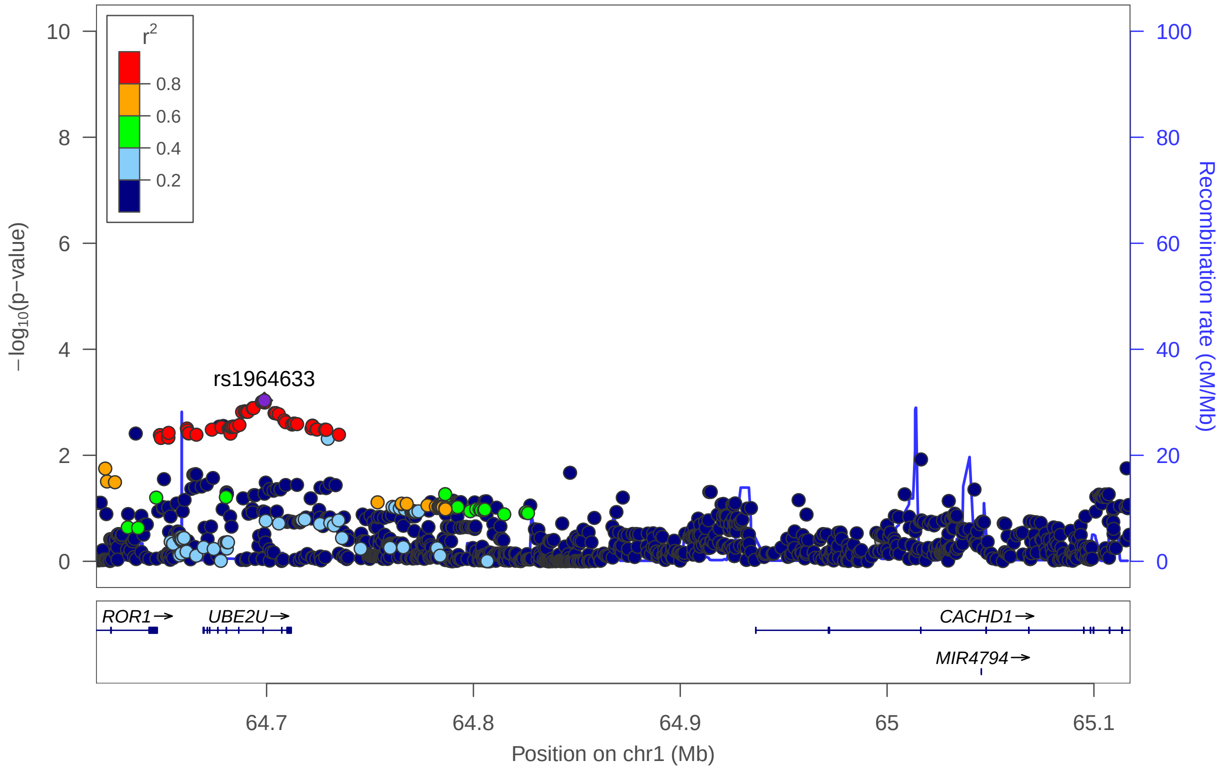

Supplementary Figure 16: **Regional association and linkage disequilibrium plots for association analysis conditioning on the top variant at the chromosome 1p31.3 susceptibility locus for AML.** Regional association plot showing the chromosome 1p31.3 AML susceptibility locus conditioning on rs10789158. For each GWAS, association tests were performed for all AML cases assuming an additive genetic model, with nominally significant principal components included in the analysis as covariates. Association summary statistics were combined for variants common to all four GWAS, in fixed effects models using PLINK. SNP coordinates based on genomic build b37/h19 are shown on the x-axis and $-log_{10}$ (P values) on the y-axis. SNPs are coloured according to their linkage disequilibrium (pairwise r^2) with the lead SNP (annotated) based on the 1000 Genomes European panel. Reference genes in the region are shown in the lower panel, with arrows indicating transcript direction, dense blocks representing exons and horizontal lines representing introns. All statistical tests were two-sided and no adjustments were made for multiple comparisons.

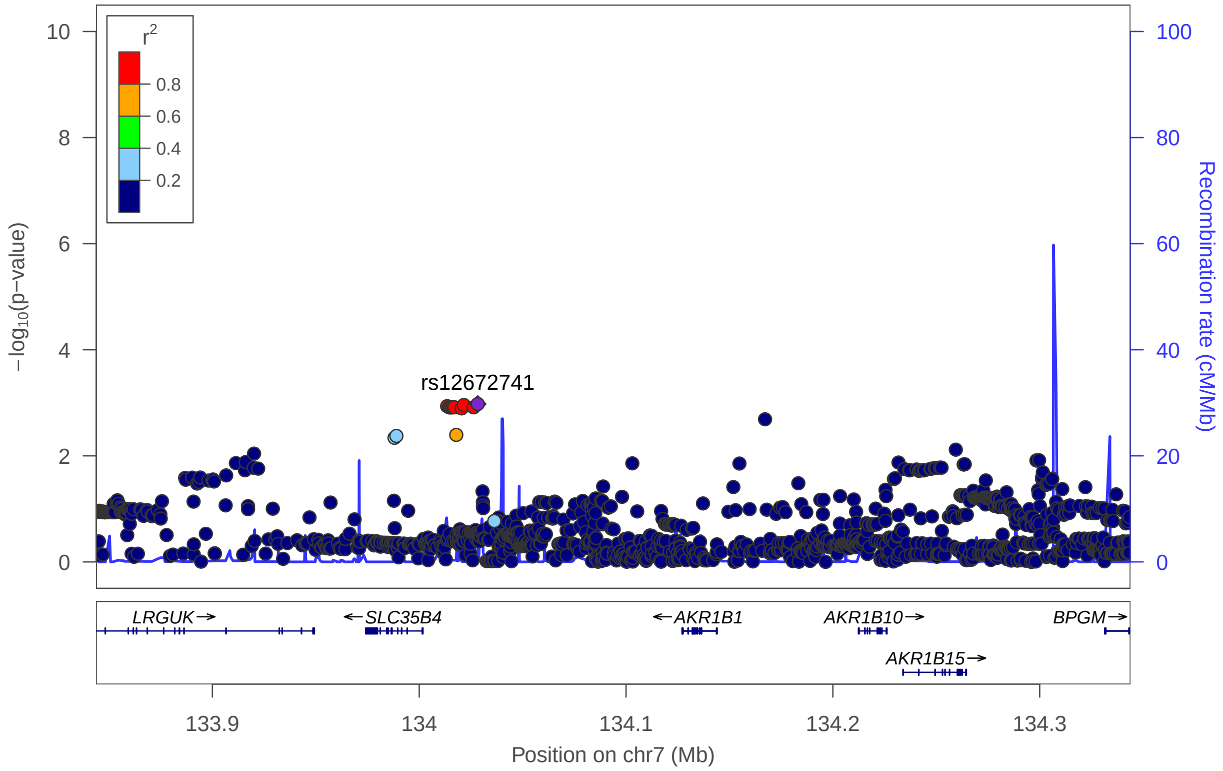

Supplementary Figure 17: **Regional association and linkage disequilibrium plots for association analysis conditioning on the top variant at the chromosome 7q33 susceptibility locus for cytogenetically normal AML.** Regional association plot showing the chromosome 7q33 AML susceptibility locus conditioning on rs17773014. For each GWAS, association tests were performed for all AML cases assuming an additive genetic model, with nominally significant principal components included in the analysis as covariates. Association summary statistics were combined for variants common to all four GWAS, in fixed effects models using PLINK. SNP coordinates based on genomic build b37/h19 are shown on the x-axis and $-log_{10}$ (P values) on the y-axis. SNPs are coloured according to their linkage disequilibrium (pairwise r^2) with the lead SNP (annotated) based on the 1000 Genomes European panel. Reference genes in the region are shown in the lower panel, with arrows indicating transcript direction, dense blocks representing exons and horizontal lines representing introns. All statistical tests were two-sided and no adjustments were made for multiple comparisons.

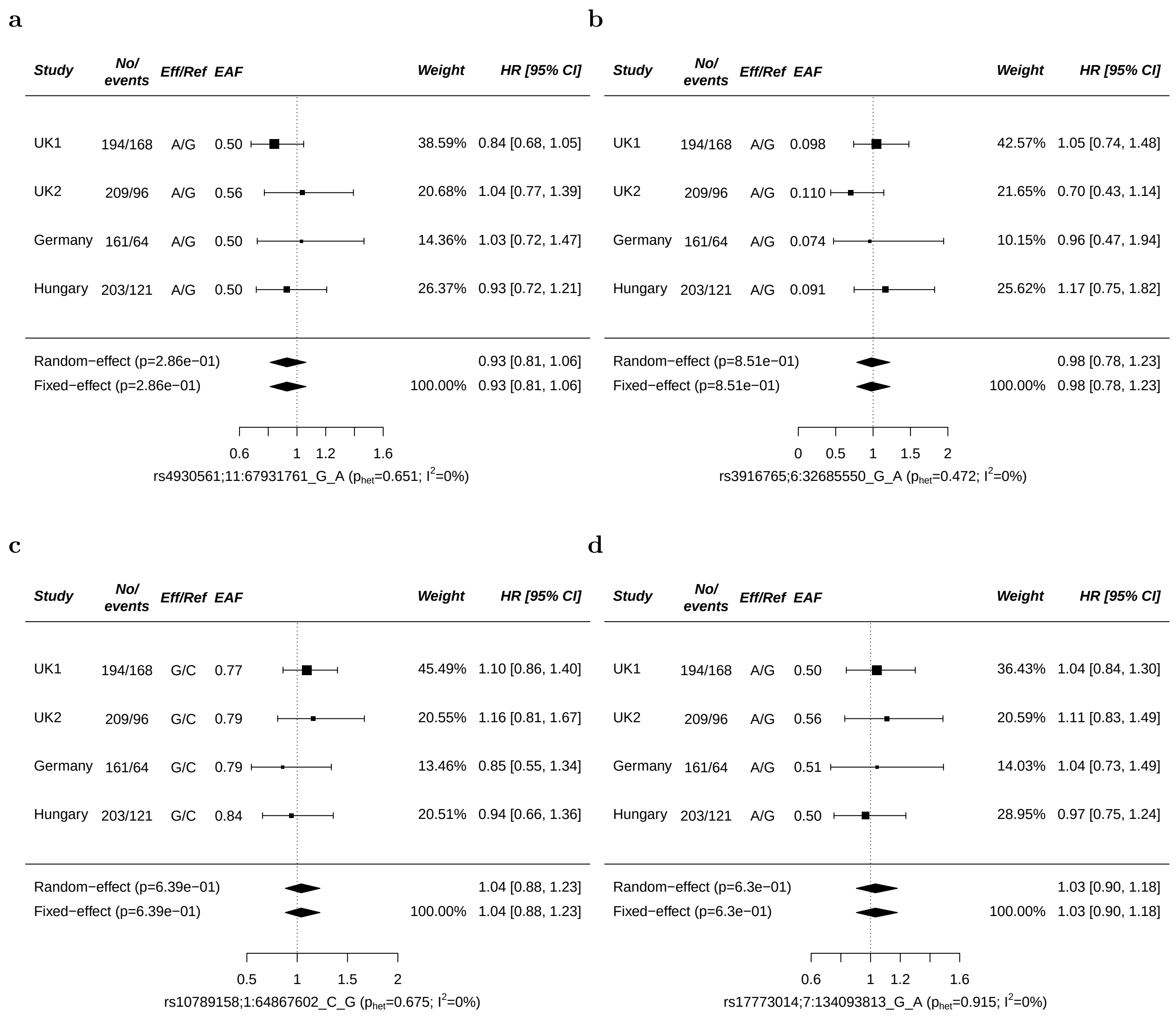

Supplementary Figure 18: **SNP effects on AML overall survival (OS) by study.** a rs4930561 b rs3916765 c rs10789158 d rs17773014. Study cohorts (UK1, UK2, Germany and Hungary), number of AML cases (cases), events, effect (Eff) and reference (Ref) allele, effect allele frequencies (EAF) and estimated hazard ratios (HR). The vertical line corresponds to the null hypothesis (HR=1). The horizontal lines and square brackets indicate 95% confidence intervals (95% CI). Areas of the boxes are proportional to the weight of the study. Diamonds represent combined estimates for fixed-effect and random-effect analysis. Cochran's Q statistic was used to test for heterogeneity such that $P_{HET} > 0.05$ indicates the presence of non-significant heterogeneity. The heterogeneity index, I^2 (0-100) was also measured which quantifies the proportion of the total variation due to heterogeneity. Overall survival was defined as the time from diagnosis to the date of last follow-up or death (event) from any cause. Cox regression analysis was used to estimate allele specific hazard ratios and 95% CIs.

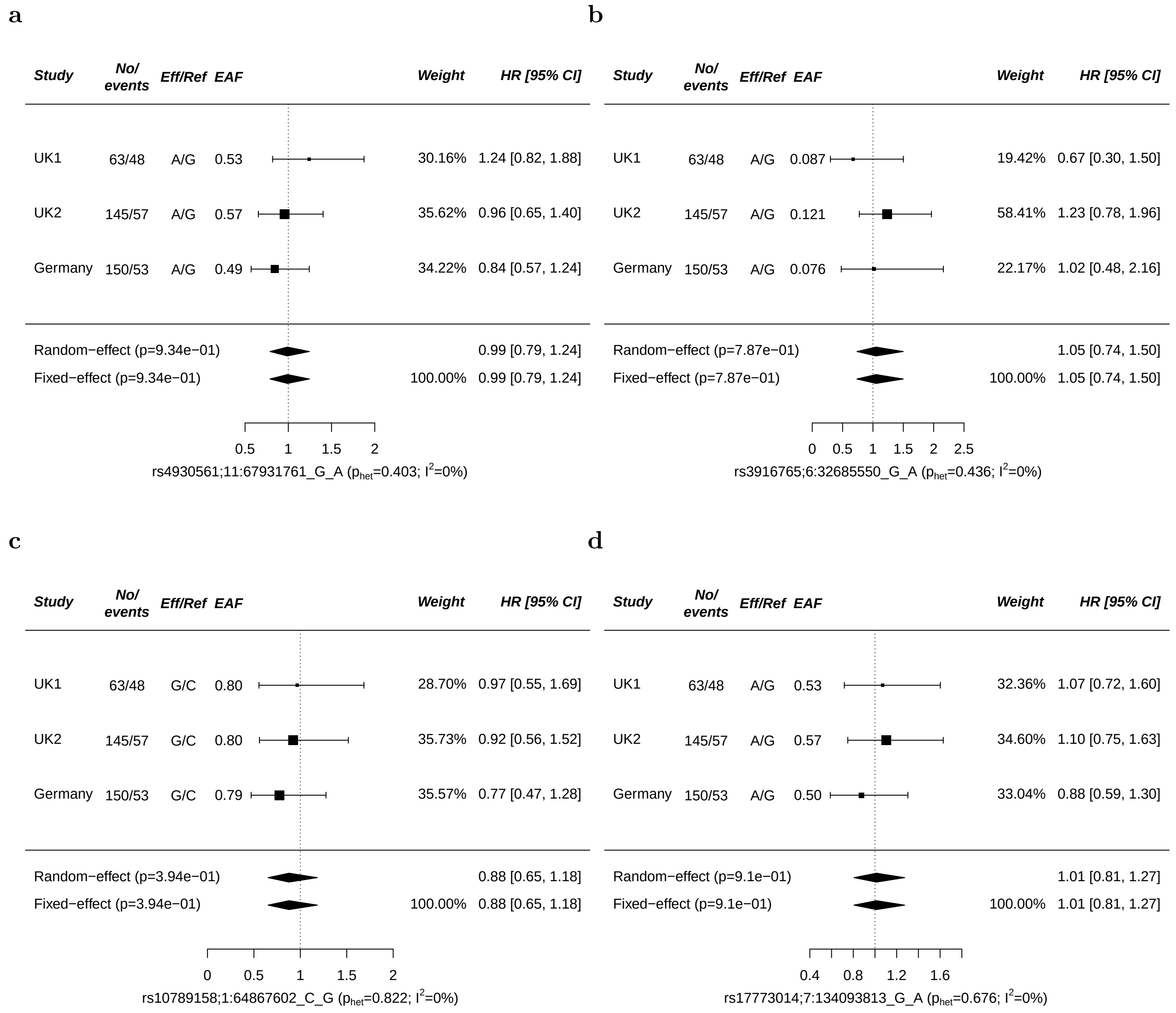

Supplementary Figure 19: **SNP effects on AML relapse-free survival (RFS) by study.** a rs4930561 b rs3916765 c rs10789158 d rs17773014. Study cohorts (UK1, UK2, Germany), number of AML cases (cases), events, effect (Eff) and reference (Ref) allele, effect allele frequencies (EAF) and estimated hazard ratios (HR). The vertical line corresponds to the null hypothesis (HR=1). The horizontal lines and square brackets indicate 95% confidence intervals (95% CI). Areas of the boxes are proportional to the weight of the study. Diamonds represent combined estimates for fixed-effect and random-effect analysis. Cochran's Q statistic was used to test for heterogeneity such that $P_{HET} > 0.05$ indicates the presence of non-significant heterogeneity. The heterogeneity index, I^2 (0-100) was also measured which quantifies the proportion of the total variation due to heterogeneity. Relapse-free survival was defined as the time from first remission to the date of last follow-up or relapse (event). Cox regression analysis was used to estimate allele specific hazard ratios and 95% CIs.

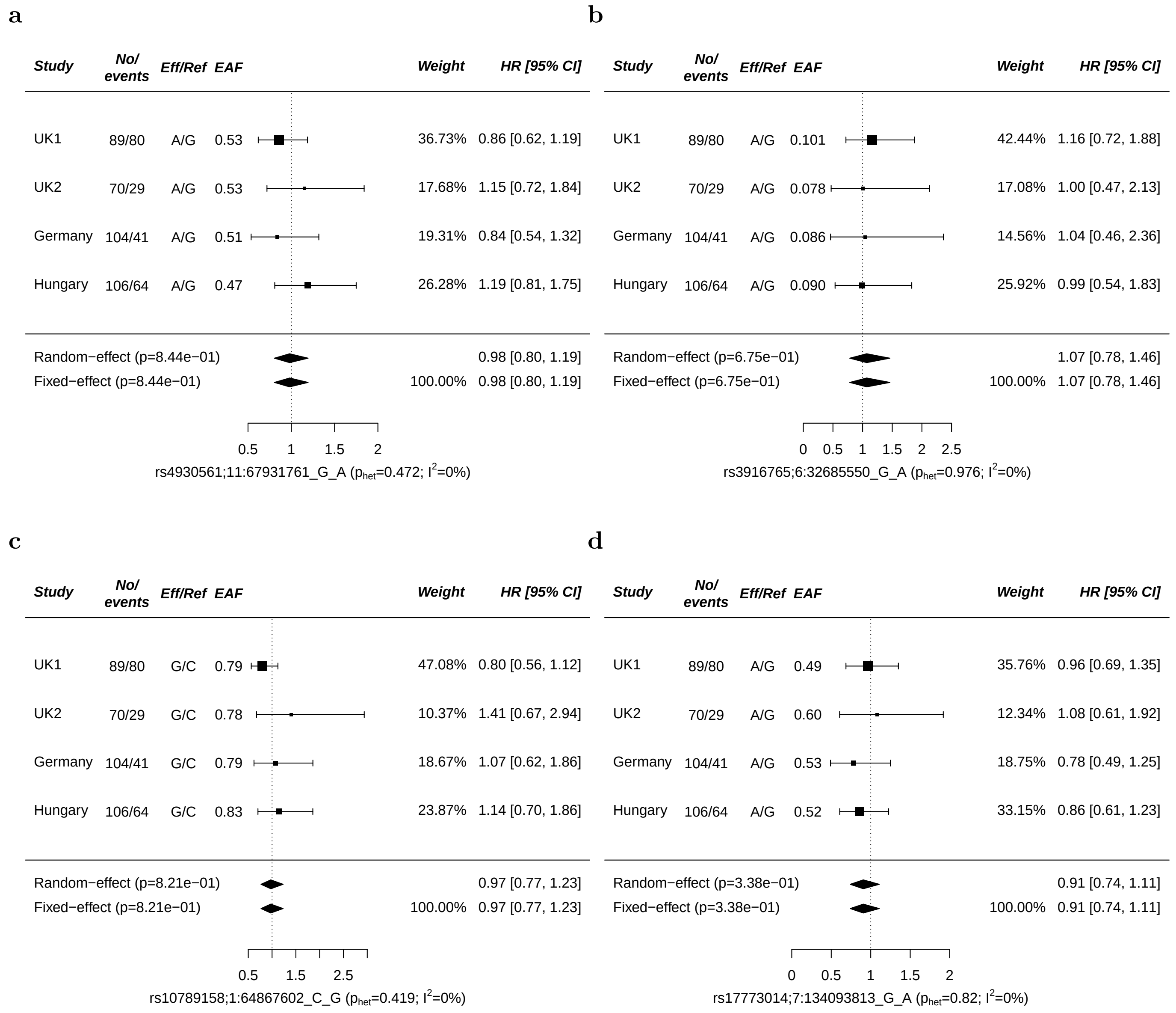

Supplementary Figure 20: **SNP effects on AML overall survival (OS) by study in cytogenetically normal AML.** a rs4930561 b rs3916765 c rs10789158 d rs17773014. Study cohorts (UK1, UK2, Germany and Hungary), number of AML cases (cases), events, effect (Eff) and reference (Ref) allele, effect allele frequencies (EAF) and estimated hazard ratios (HR). The vertical line corresponds to the null hypothesis (HR=1). The horizontal lines and square brackets indicate 95% confidence intervals (95% CI). Areas of the boxes are proportional to the weight of the study. Diamonds represent combined estimates for fixed-effect and random-effect analysis. Cochran's Q statistic was used to test for heterogeneity such that $P_{HET} > 0.05$ indicates the presence of non-significant heterogeneity. The heterogeneity index, I^2 (0-100) was also measured which quantifies the proportion of the total variation due to heterogeneity. Overall survival was defined as the time from diagnosis to the date of last follow-up or death (event) from any cause. Cox regression analysis was used to estimate allele specific hazard ratios and 95% CIs.

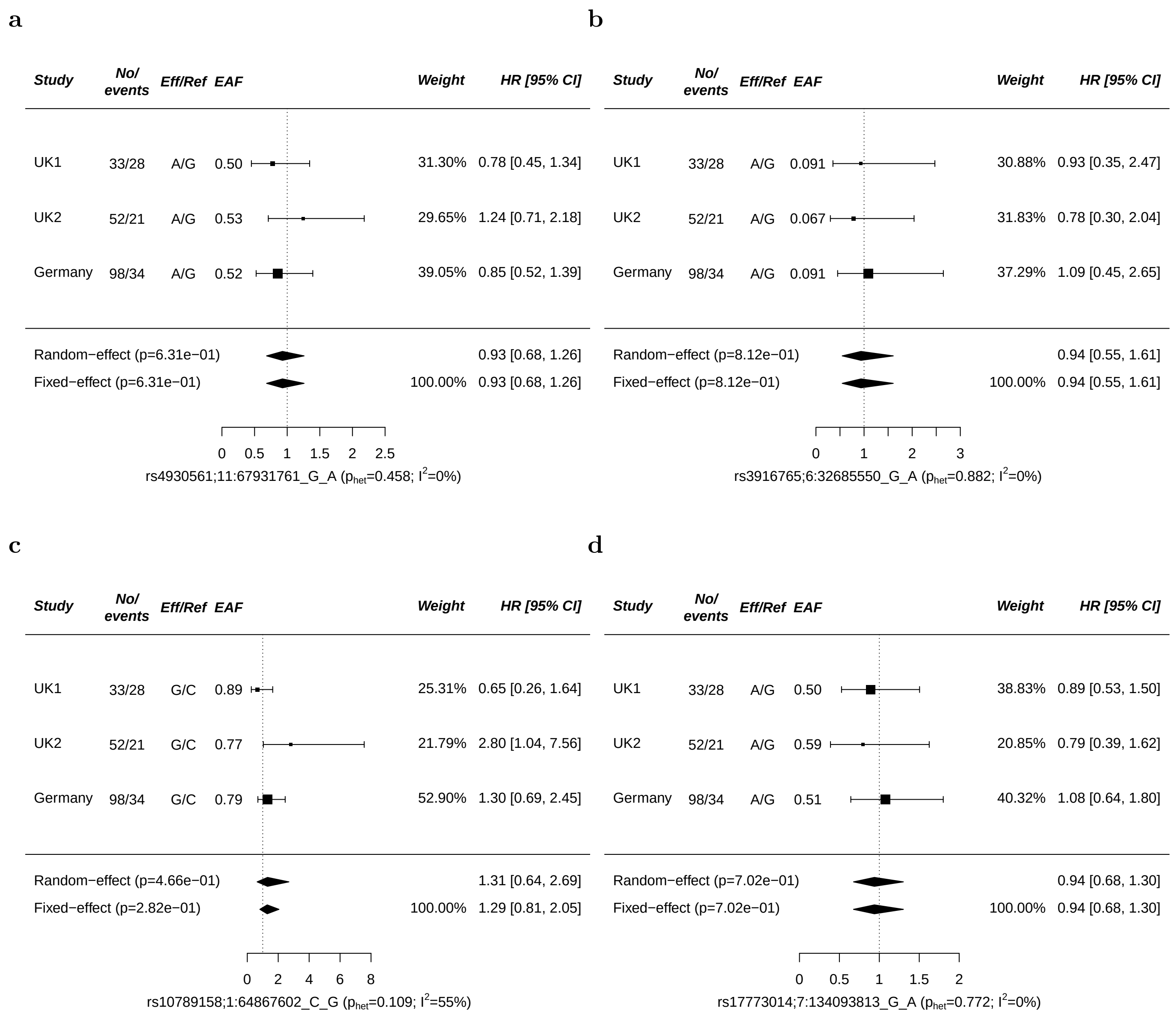

Supplementary Figure 21: **SNP effects on AML relapse-free survival (RFS) by study in cytogenetically normal AML.** a rs4930561 b rs3916765 c rs10789158 d rs17773014. Study cohorts (UK1, UK2, Germany), number of AML cases (cases), events, effect (Eff) and reference (Ref) allele, effect allele frequencies (EAF) and estimated hazard ratios (HR). The vertical line corresponds to the null hypothesis (HR=1). The horizontal lines and square brackets indicate 95% confidence intervals (95% CI). Areas of the boxes are proportional to the weight of the study. Diamonds represent combined estimates for fixed-effect and random-effect analysis. Cochran's Q statistic was used to test for heterogeneity such that $P_{HET} > 0.05$ indicates the presence of non-significant heterogeneity. The heterogeneity index, I^2 (0-100) was also measured which quantifies the proportion of the total variation due to heterogeneity. Relapse-free survival was defined as the time from first remission to the date of last follow-up or relapse (event). Cox regression analysis was used to estimate allele specific hazard ratios and 95% CIs.

a

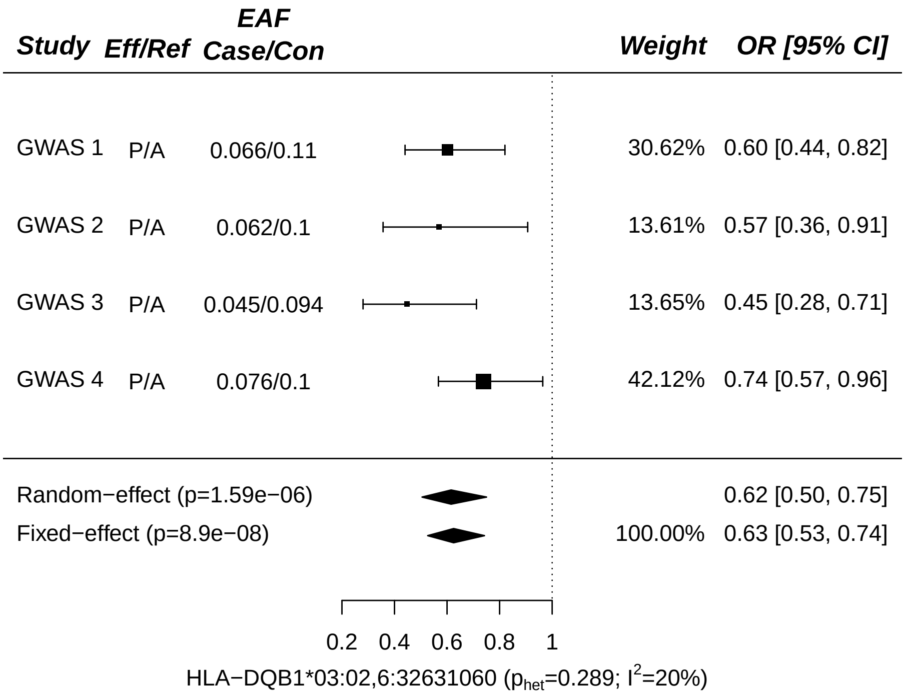

b

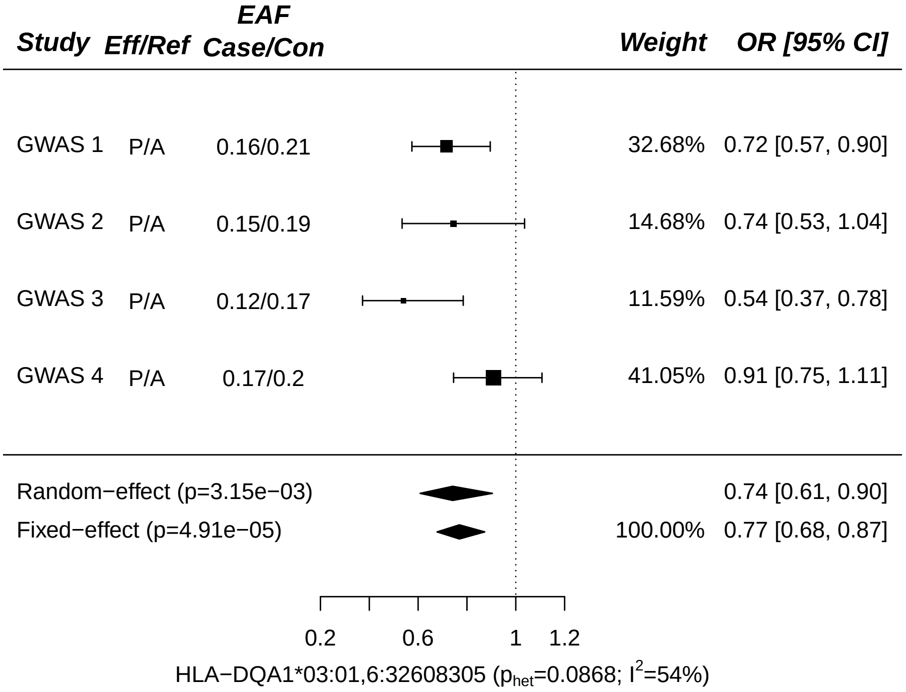

Supplementary Figure 22: **Forest plots for HLA-DQB1*03:02 and HLA-DQA1*03:01 associations with cytogenetically normal AML.** Effect allele frequencies (EAF) and estimated odds ratios (OR) for HLA-DQB1*03:02 a and HLA-DQA1*03:01 b. The vertical line corresponds to the null hypothesis (OR= 1). The horizontal lines and square brackets indicate 95% confidence intervals (95% CI). Areas of the boxes are proportional to the weight of the study. Diamonds represent combined estimates for fixedeffects and randomeffects analysis. Cochran's Q statistic was used to test for heterogeneity such that $P_{HET} > 0.05$ indicates the presence of non-significant heterogeneity. The heterogeneity index I^2 (0-100) was also measured which quantifies the proportion of the total variation due to heterogeneity. All statistical tests were two-sided and no adjustments were made for multiple comparisons. P, present; A, absent; Eff, effect; Ref, reference.

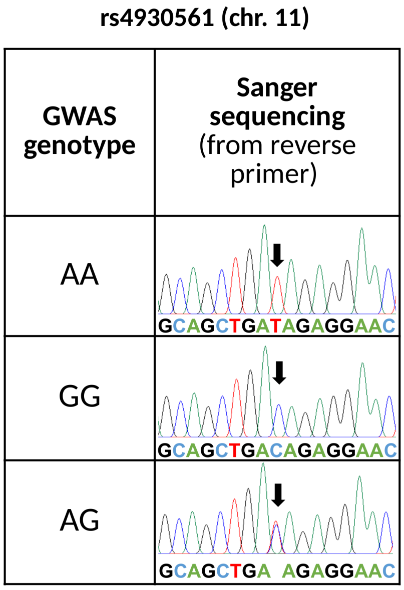

Supplementary Figure 23: **Representative genotype results for rs4930561.** Sanger sequencing was successful for 124 AML cases with 100% (124/124) concordance between GWAS genotyping and Sanger sequencing. Primer sequences for rs4930561 were 5'CCGATTCTTCTGGGGCTTGT3' (forward) and 5'TCTGCAGCATGATTGGAGCA3' (reverse).

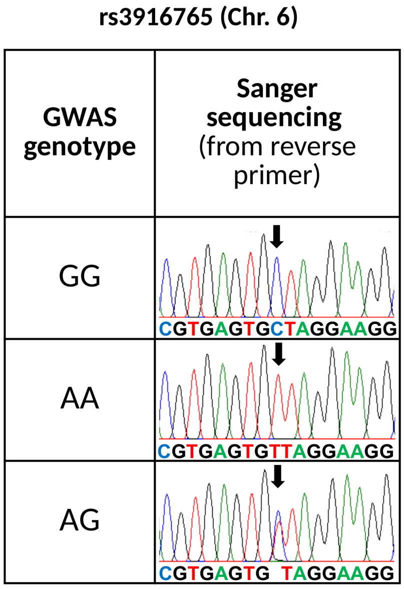

Supplementary Figure 24: **Representative genotype results for rs3916765.** Sanger sequencing was successful for 139 AML cases with 100% (139/139) concordance between GWAS genotyping and Sanger sequencing. Primer sequences for rs3916765 were 5'TTGGTACCTGGGGTATGCTGAA3' (forward) and 5'TGGAGGCTGCCTTGAGATACTA3'(reverse).

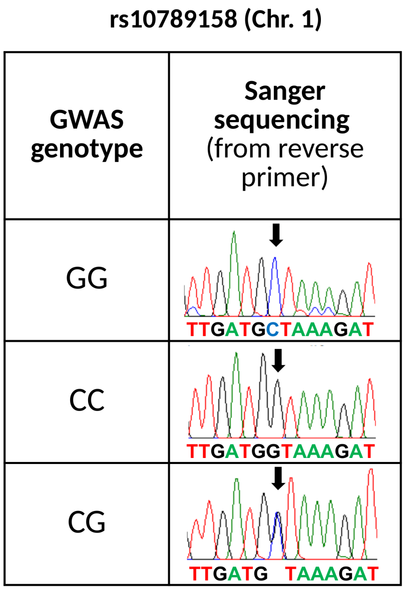

Supplementary Figure 25: **Representative genotype results for rs10789158.** Sanger sequencing was successful for 130 AML cases with 98.5% (128/130) concordance between GWAS genotyping and Sanger sequencing. Primer sequences for rs10789158 were 5'AGCACGTTACAGACTATGCCT3' (forward) and 5'AGCTCAAAGACATGGGGCAA3' (reverse).

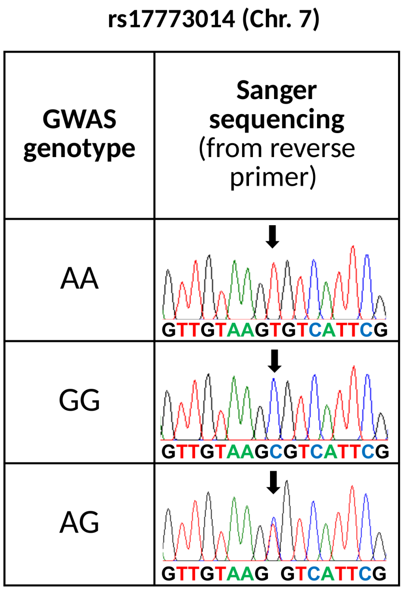

Supplementary Figure 26: **Representative genotype results for rs17773014.** Sanger sequencing was successful for 120 AML cases with 99.2% (119/120) concordance between GWAS genotyping and Sanger sequencing. Primer sequences for rs17773014 were 5'TGTATAACCAAGGGACCGCAC3' (forward) and 5'CACCCCGTCCCATATCCAATG3' (reverse).

SUPPLEMENTARY TABLES

Genome-wide association study identifies susceptibility loci for acute myeloid leukemia

Lin *et al*

Supplementary Table 1 –Acute myeloid leukemia (AML) case and control characteristics

N, number; GWAS, genome wide association study; APL, acute promyelocytic leukemia; CBF, core binding factor (CBF AML is defined as t(8;21) or inv(16)); Complex karyotype is defined as 3 or more independent alterations; Monosomal karyotype is defined as the presence of 2 or more autosomal monosomies or a single autosomal monosomy with at least one structural abnormality. For some cases age and/or gender was not declared or not known. Some cytogenetic sub-groups are not mutually exclusive.

	**GWAS 1**		**GWAS 2**		**GWAS 3**		**GWAS 4**	
	**Cases**	**Controls**	**Cases**	**Controls**	**Cases**	**Controls**	**Cases**	**Controls**
**Total, N**	1119	2671	931	2477	991	1612	977	3728
**Age, years**								
Median (range)	54 (13-91)	45 (45-45)	49 (<1-95)	45 (15-65)	60 (12-93)	62 (32-81)	63 (<1-93)	57 (40-70)
**Sex, N (%)**								
Males	605 (54)	1377 (50)	486 (52)	1225 (49)	500 (50)	785 (49)	511 (52)	1746 (47)
Females	514 (46)	1294 (50)	444 (48)	1252 (51)	478 (48)	827 (51)	466 (48)	1982 (53)
Not known	0 (0)	0 (0)	1 (0)	0 (0)	13 (2)	0 (0)	0 (0)	0 (0)
**Cytogenetics, N (%)**								
Successful cytogenetics	879 (100)	-	654 (100)	-	666 (100)	-	718 (100)	-
Normal cytogenetics	359 (41)	-	177 (27)	-	286 (43)	-	465 (65)	-
Abnormal cytogenetics	520 (59)	-	477 (73)	-	380 (57)	-	253 (35)	-
APL t(15.17)	131 (15)	-	380 (58)	-	108 (16)	-	38 (5)	-
CBF AML	72 (8)	-	50 (8)	-	219 (33)	-	27 (4)	-
del5.del7	65 (7)	-	58 (9)	-	68 (10)	-	57 (8)	-
Complex (3 or more)	69 (8)	-	61 (9)	-	89 (13)	-	82 (11)	-
Any translocation	241 (27)	-	459 (70)	-	253 (38)	-	105 (15)	-
Any trisomy	106 (12)	-	70 (11)	-	55 (8)	-	104 (14)	-
Any monosomy	54 (6)	-	56 (9)	-	67 (10)	-	62 (9)	-
Monosomal karyotype	20 (2)	-	34 (5)	-	54 (8)	-	46 (6)	-

Supplementary Table 2 – SNPs showing evidence of an association with risk of acute myeloid leukemia (AML) at $P \leq 10^{-6}$.

Association tests were performed for all AML cases and cytogenetically normal AML cases assuming an additive genetic model, with nominally significant principal components included in the analysis as covariates. Association summary statistics were combined for variants common to GWAS 1, GWAS 2 and GWAS 3, in fixed effects models using PLINK. All statistical tests were two-sided and no adjustments were made for multiple comparisons. Results are based on meta-analysis of GWAS 1, GWAS 2 and GWAS 3. SNP, single nucleotide polymorphism; Chr, chromosome; OR, odds ratio; Q, Cochran's Q statistic; I^2, heterogeneity index I^2. [a]hg19 coordinates. All statistical tests were two sided.

SNP	AML karotype	Chr	Position[a]	Chr band	Allele 1	Allele 2	Meta *P* value (fixed effect)	Meta P (random effect)	Meta OR (fixed effect)	Meta OR (random effect)	Q	I^2
rs4674579	All AML	2	222167434	2q36.1	C	T	7.00E-07	7.00E-07	0.8063	0.8063	0.63	0
rs2621279	All AML	3	158940084	3q25.32	A	G	4.63E-07	4.63E-07	0.815	0.815	0.7933	0
rs13164987	All AML	5	168247045	5q35.1	C	T	4.79E-07	4.79E-07	0.7885	0.7885	0.6976	0
rs13183143	All AML	5	168247046	5q35.1	A	G	4.37E-07	4.37E-07	0.7876	0.7876	0.6849	0
rs11481	All AML	11	67820335	11q13.2	A	T	8.04E-07	8.04E-07	1.2044	1.2044	0.4994	0
rs10896298	All AML	11	67931459	11q13.2	T	C	9.83E-07	9.83E-07	1.1777	1.1777	0.4718	0
rs4930561	All AML	11	67931761	11q13.2	A	G	9.26E-07	9.26E-07	1.1781	1.1781	0.468	0
rs6056038	All AML	20	8701468	20p12.3	C	T	8.90E-07	8.90E-07	0.762	0.762	0.9602	0
rs6056041	All AML	20	8702867	20p12.3	C	T	5.41E-07	5.41E-07	0.7569	0.7569	0.9746	0
rs6077414	All AML	20	8704552	20p12.3	C	T	3.77E-07	3.77E-07	0.7533	0.7533	0.9702	0
rs6056043	All AML	20	8705496	20p12.3	C	A	3.94E-07	3.94E-07	0.7534	0.7534	0.9756	0
rs75391980	Normal	4	96948521	4q22.3	C	T	3.40E-08	3.40E-08	1.7457	1.7457	0.4139	0
rs9275092	Normal	6	32648987	6p21.32	T	C	6.47E-07	6.47E-07	0.5511	0.5511	0.566	0
rs9275095	Normal	6	32649088	6p21.32	G	C	5.48E-07	5.48E-07	0.5456	0.5456	0.5911	0
rs9275097	Normal	6	32649126	6p21.32	G	A	4.48E-07	4.48E-07	0.5486	0.5486	0.5575	0
rs9275098	Normal	6	32649161	6p21.32	T	C	4.88E-07	4.88E-07	0.553	0.553	0.5503	0
rs9275167	Normal	6	32653263	6p21.32	G	A	4.94E-07	4.94E-07	0.5616	0.5616	0.6229	0
rs9275184	Normal	6	32654714	6p21.32	C	T	5.22E-07	5.22E-07	0.5631	0.5631	0.6254	0
rs9275203	Normal	6	32656947	6p21.32	A	G	4.92E-07	4.92E-07	0.5623	0.5623	0.6321	0
rs9275207	Normal	6	32657710	6p21.32	G	A	4.36E-07	4.36E-07	0.5603	0.5603	0.6523	0
rs9275213	Normal	6	32658335	6p21.32	C	T	4.39E-07	4.39E-07	0.5605	0.5605	0.6568	0

rs9275214	Normal	6	32658665	6p21.32	A	G	4.47E-07	4.47E-07	0.5606	0.5606	0.6594	0
rs9275221	Normal	6	32659099	6p21.32	C	T	4.62E-07	4.62E-07	0.5609	0.5609	0.6583	0
rs9275223	Normal	6	32659839	6p21.32	A	G	4.68E-07	4.68E-07	0.5611	0.5611	0.6645	0
rs9275275	Normal	6	32662607	6p21.32	A	G	4.94E-07	4.94E-07	0.5616	0.5616	0.6731	0
rs9275293	Normal	6	32663308	6p21.32	C	T	5.06E-07	5.06E-07	0.5619	0.5619	0.6777	0
rs9275296	Normal	6	32663431	6p21.32	C	T	5.08E-07	5.08E-07	0.562	0.562	0.6791	0
rs9275300	Normal	6	32664126	6p21.32	G	A	4.47E-07	4.47E-07	0.5598	0.5598	0.6864	0
rs9275307	Normal	6	32664990	6p21.32	T	A	5.11E-07	5.11E-07	0.562	0.562	0.678	0
rs9275308	Normal	6	32665255	6p21.32	A	T	5.10E-07	5.10E-07	0.5616	0.5616	0.6714	0
rs9275310	Normal	6	32665319	6p21.32	G	T	4.96E-07	4.96E-07	0.5614	0.5614	0.6725	0
rs9275311	Normal	6	32665640	6p21.32	T	G	4.97E-07	4.97E-07	0.5615	0.5615	0.6729	0
rs9275313	Normal	6	32665759	6p21.32	T	G	4.25E-07	4.25E-07	0.5594	0.5594	0.662	0
rs9275314	Normal	6	32665909	6p21.32	A	C	5.17E-07	5.17E-07	0.5618	0.5618	0.6682	0
rs9275315	Normal	6	32665912	6p21.32	A	C	5.17E-07	5.17E-07	0.5618	0.5618	0.6687	0
rs9275324	Normal	6	32666635	6p21.32	T	C	4.51E-07	4.51E-07	0.5577	0.5577	0.6839	0
rs9275325	Normal	6	32666651	6p21.32	T	G	4.97E-07	4.97E-07	0.5585	0.5585	0.6767	0
rs9275326	Normal	6	32666660	6p21.32	T	C	5.05E-07	5.05E-07	0.5588	0.5588	0.6767	0
rs9275334	Normal	6	32667107	6p21.32	C	T	5.06E-07	5.06E-07	0.5616	0.5616	0.6756	0
rs9275373	Normal	6	32668411	6p21.32	A	G	2.40E-07	2.40E-07	0.5639	0.5639	0.4842	0
rs9275382	Normal	6	32668831	6p21.32	C	T	2.54E-07	2.54E-07	0.5419	0.5419	0.8059	0
rs9275383	Normal	6	32668846	6p21.32	T	G	2.49E-07	2.49E-07	0.5483	0.5483	0.7753	0
rs9275387	Normal	6	32669013	6p21.32	C	T	5.18E-07	5.18E-07	0.5619	0.5619	0.6764	0
rs9275394	Normal	6	32669454	6p21.32	C	A	5.12E-07	5.12E-07	0.5616	0.5616	0.6776	0
rs9275395	Normal	6	32669483	6p21.32	G	A	5.10E-07	5.10E-07	0.5615	0.5615	0.6777	0
rs9275400	Normal	6	32669761	6p21.32	G	C	5.16E-07	5.16E-07	0.5619	0.5619	0.6768	0
rs9275422	Normal	6	32670548	6p21.32	A	G	5.03E-07	5.03E-07	0.5616	0.5616	0.6713	0
rs9275426	Normal	6	32670912	6p21.32	C	T	5.10E-07	5.10E-07	0.5615	0.5615	0.6725	0
rs9275429	Normal	6	32671057	6p21.32	A	G	5.11E-07	5.11E-07	0.5616	0.5616	0.6756	0
rs9275430	Normal	6	32671086	6p21.32	T	C	5.13E-07	5.13E-07	0.5617	0.5617	0.674	0
rs9275434	Normal	6	32671247	6p21.32	T	C	5.13E-07	5.13E-07	0.5617	0.5617	0.6709	0

rs9275435	Normal	6	32671332	6p21.32	C	G	5.12E-07	5.12E-07	0.5616	0.5616	0.6704	0
rs9275437	Normal	6	32671412	6p21.32	T	C	5.13E-07	5.13E-07	0.5616	0.5616	0.6703	0
rs9275442	Normal	6	32671755	6p21.32	A	G	5.09E-07	5.09E-07	0.5616	0.5616	0.6708	0
rs9275476	Normal	6	32672624	6p21.32	C	T	5.40E-07	5.40E-07	0.5615	0.5615	0.6702	0
rs9275477	Normal	6	32672641	6p21.32	C	A	5.74E-07	5.74E-07	0.5516	0.5516	0.721	0
rs9275486	Normal	6	32673099	6p21.32	A	T	5.34E-07	5.34E-07	0.5621	0.5621	0.6669	0
rs9275490	Normal	6	32673385	6p21.32	G	C	5.37E-07	5.37E-07	0.5622	0.5622	0.6672	0
rs9275495	Normal	6	32673574	6p21.32	T	A	5.59E-07	5.59E-07	0.5626	0.5626	0.6731	0
rs9275503	Normal	6	32673931	6p21.32	G	A	6.37E-07	6.37E-07	0.5566	0.5566	0.7228	0
rs9275530	Normal	6	32675523	6p21.32	C	G	5.52E-07	5.52E-07	0.5627	0.5627	0.6811	0
rs9275532	Normal	6	32675634	6p21.32	G	C	5.47E-07	5.47E-07	0.5625	0.5625	0.6785	0
rs9275541	Normal	6	32676159	6p21.32	G	C	5.40E-07	5.40E-07	0.5623	0.5623	0.6747	0
rs4273728	Normal	6	32678491	6p21.32	C	T	4.82E-07	4.82E-07	0.5606	0.5606	0.6383	0
rs9275583	Normal	6	32680299	6p21.32	A	G	4.18E-07	4.18E-07	0.5587	0.5587	0.6045	0
rs9275592	Normal	6	32680620	6p21.32	T	G	4.16E-07	4.16E-07	0.5586	0.5586	0.5971	0
rs7454108	Normal	6	32681483	6p21.32	C	T	3.77E-07	3.77E-07	0.5575	0.5575	0.5786	0
rs3957146	Normal	6	32681530	6p21.32	C	T	3.76E-07	3.76E-07	0.5575	0.5575	0.5777	0
rs3998159	Normal	6	32682019	6p21.32	C	A	5.21E-07	5.21E-07	0.5625	0.5625	0.5693	0
rs3957148	Normal	6	32682137	6p21.32	G	A	3.15E-07	3.15E-07	0.5546	0.5546	0.6094	0
rs9275599	Normal	6	32682429	6p21.32	T	C	4.58E-08	4.58E-08	0.5071	0.5071	0.6565	0
rs3997854	Normal	6	32682915	6p21.32	G	T	4.18E-09	2.78E-08	0.5204	0.5172	0.3289	10.06
rs3873448	Normal	6	32683055	6p21.32	T	C	8.51E-09	9.63E-06	0.5296	0.5117	0.1801	41.67
rs3873453	Normal	6	32683382	6p21.32	T	C	3.95E-07	1.15E-05	0.5858	0.5758	0.2628	25.17
rs9275607	Normal	6	32683645	6p21.32	G	A	1.10E-07	1.10E-07	0.5883	0.5883	0.6732	0
rs9275610	Normal	6	32683750	6p21.32	C	T	7.52E-08	7.52E-08	0.5838	0.5838	0.6345	0
rs9275611	Normal	6	32683763	6p21.32	A	G	2.01E-08	1.82E-06	0.5405	0.5289	0.252	27.45
rs9275613	Normal	6	32684105	6p21.32	T	C	2.07E-08	1.47E-06	0.5444	0.5335	0.259	25.98
rs9275614	Normal	6	32684257	6p21.32	G	A	2.11E-08	1.89E-06	0.5451	0.5334	0.2516	27.54
rs9275615	Normal	6	32684272	6p21.32	A	T	2.13E-08	1.81E-06	0.5452	0.5337	0.253	27.25
rs6916779	Normal	6	32684317	6p21.32	C	T	8.43E-08	8.43E-08	0.5888	0.5888	0.6183	0

rs9275618	Normal	6	32684387	6p21.32	G	A	2.17E-08	1.64E-06	0.5455	0.5343	0.2566	26.49
rs9275638	Normal	6	32684760	6p21.32	T	C	1.30E-07	1.30E-07	0.5861	0.5861	0.5088	0
rs3916765	Normal	6	32685550	6p21.32	A	G	8.53E-09	2.14E-07	0.5327	0.525	0.2939	18.33
rs10969985	Normal	9	30943593	9p21.1	T	C	6.52E-07	0.00471	2.1281	1.9632	0.1071	55.25
rs143280565	Normal	13	63147291	13q21.31	T	G	1.01E-07	1.38E-05	2.793	2.8335	0.2212	33.72

Supplementary Table 3 – Risk loci for acute myeloid leukemia (AML) stratified by age. Table shows fixed effects meta odds ratios for risk loci at 11q13.2 (rs4930561), 1p31.3 (rs10789158), 6p21.32 (rs3916765) and 7q33 (rs17773014) stratified by age. Cases and controls were stratified into those < 55 years and ≥ 55 years. GWAS 1 was not included in the meta-analysis for the ≥ 55 age group because the controls were recruited to the 1958 Birth Cohort and were all genotyped at the age of 45 years. For some cases age was not declared or not known. For each GWAS, association tests were performed for all AML cases and cytogenetically normal AML cases assuming an additive genetic model, with nominally significant principal components included in the analysis as covariates. Association summary statistics were combined for variants common to all four GWAS for < 55 year olds and common to GWAS 2, GWAS 3 and GWAS 4 for ≥ 55 year olds, in fixed effects models using PLINK. All statistical tests were two-sided and no adjustments were made for multiple comparisons. N, number; OR, odds ratio; 95% CI, 95% confidence interval; Q, Cochran's Q statistic; I^2, heterogeneity index I^2; *P*, *P* value.

AML risk variant	Cytogenetics	Age (years)	Case, N / Control, N	GWAS, N	OR (95% CI)	*P* value	I^2	*P*Q
rs4930561	All AML	All ages	4018/10488	4	1.17 (1.11-1.24)	2.15e-08	0.00	0.665
		< 55	1401/7019	4	1.21 (1.11-1.32)	3.0e-05	10.07	0.34
		≥ 55	1222/3469	3	1.10 (0.99-1.22)	0.08	0.00	0.76
					Test for heterogeneity		46.87	0.17
rs10789158	All AML	All ages	4018/10488	4	1.22 (1.13-1.31)	2.25e-07	0.00	0.422
		< 55	1401/7019	4	1.22 (1.09-1.38)	8.8e-04	0.00	0.82
		≥ 55	1222/3469	3	1.10 (0.95-1.27)	0.19	0.00	0.74
					Test for heterogeneity		16.99	0.27
rs3916765	Normal	All ages	1287/10488	4	1.72 (1.46-2.03)	1.51e-10	26	0.256
		< 55	480/7019	4	1.64 (1.25-2.16)	3.5e-04	0.00	1.00
		≥ 55	518/3470	3	1.66 (1.27-2.17)	2.1e-04	43.90	0.17
					Test for heterogeneity		0.00	0.95
rs17773014	Normal	All ages	1287/10488	4	1.26 (1.15-1.37)	4.09e-07	15	0.315
		< 55	480/7019	4	1.36 (1.18-1.58)	4.3e-05	50.84	0.11
		≥ 55	518/3470	3	1.18 (1.03-1.37)	0.02	59.09	0.09
					Test for heterogeneity		50.10	0.16

Supplementary Table 4 – Risk loci for acute myeloid leukemia (AML) stratified by sex. Table shows fixed effects meta odds ratios for risk loci at 11q13.2 (rs4930561), 1p31.3 (rs10789158), 6p21.32 (rs3916765) and 7q33 (rs17773014) stratified by sex. For some cases sex was not declared or not known. For each GWAS, association tests were performed for all AML cases and cytogenetically normal AML cases assuming an additive genetic model, with nominally significant principal components included in the analysis as covariates. Association summary statistics were combined for variants common to all four GWAS, in fixed effects models using PLINK. All statistical tests were two-sided and no adjustments were made for multiple comparisons. N, number; OR, odds ratio; 95% CI, 95% confidence interval; Q, Cochran's Q statistic; I^2, heterogeneity index I^2; *P*, *P* value.

AML risk variant	Cytogenetics	Sex	Case, N / Control, N	GWAS, N	OR (95% CI)	*P* value	I^2	*P*Q
rs4930561	All AML	All	4018/10488	4	1.17 (1.11-1.24)	2.15e-08	0.00	0.665
		Males	2102/5133	4	1.18 (1.09-1.27)	4.5e-05	28.77	0.24
		Females	1902/5255	4	1.17 (1.08-1.27)	1.1e-04	0.00	0.67
					Test for heterogeneity		0.00	0.88
rs10789158	All AML	All	4018/10488	4	1.22 (1.13-1.31)	2.25e-07	0.00	0.422
		Males	2102/5133	4	1.27 (1.14-1.41)	9.8e-06	0.00	0.83
		Females	1902/5255	4	1.18 (1.06-1.31)	2.4e-03	36.69	0.19
					Test for heterogeneity		0.00	0.34
rs3916765	Normal	All	1287/10488	4	1.72 (1.46-2.03)	1.51e-10	26	0.256
		Males	642/5133	4	1.45 (1.16-1.80)	1.1e-03	55.10	0.08
		Females	645/5355	4	2.11 (1.63-2.73)	1.2e-08	0.00	0.86
					Test for heterogeneity		78.47	0.03
rs17773014	Normal	All	1287/10488	4	1.26 (1.15-1.37)	4.09e-07	15	0.315
		Males	642/5133	4	1.27 (1.12-1.44)	2.3e-04	0.00	0.65
		Females	645/5355	4	1.24 (1.09-1.40)	7.0e-03	4.30	0.37
					Test for heterogeneity		0.00	0.79

Supplementary Table 5 - *cis*-eQTL analysis of rs4930561 in whole blood. *cis*-eQTL data for loci annotated to within 500kb of rs4930561 derived from whole blood expression data collated by the eQTLGen Consortium (http://www.eqtlgen.org/cis-eqtls.html). SNP, single nucleotide polymorphism; QTL, expression quantitative trait loci; Chr, chromosome. [a]hg19 coordinates; [b]Unadjusted *P* value; [c]Benjamini-Hochberg corrected *P* value.

SNP	SNP Position[a]	Assessed allele eQTL	Chr	Gene	Gene Symbol	Gene Position[a]	P value eQTL[b]	P_{BH} eQTL[c]	Z score
rs4930561	67931761	G	11	ENSG00000160172	FAM86C2P	67565963	0.552111	0.763212	0.5947
				ENSG00000255306	RP5-901A4.1	67795102	1.75E-25	4.1E-24	-10.4334
				ENSG00000110717	NDUFS8	67801097	3.23E-05	0.000169	-4.1564
				ENSG00000110719	TCIRG1	67812422	1.33E-05	7.83E-05	4.3546
				ENSG00000255031	RP11-802E16.3	67819718	7.19E-16	6.76E-15	-8.0671
				ENSG00000110721	CHKA	67854618	2.21E-15	1.73E-14	7.9291
				ENSG00000239559	RPL37P2	67450386	0.954545	0.99533	0.0571
				ENSG00000132746	ALDH3B2	67439152	0.432923	0.678246	-0.7841
				ENSG00000132744	ACY3	67414078	0.19532	0.437145	-1.2949
				ENSG00000167799	NUDT8	67396405	0.46968	0.700717	-0.723
				ENSG00000231793	DOC2GP	67381926	1.96E-13	1.32E-12	-7.3514
				ENSG00000110066	KMT5B	67951812	0.966818	0.99533	0.0418
				ENSG00000167792	NDUFV1	67377164	0.966339	0.99533	0.0422
				ENSG00000084207	GSTP1	67352598	0.50605	0.720739	-0.665
				ENSG00000171067	C11orf24	68034136	0.974153	0.99533	0.0324
				ENSG00000162337	LRP5	68148410	0.853072	0.99533	-0.1852
				ENSG00000167797	CDK2AP2	67275044	0.297134	0.537126	-1.0426
				ENSG00000110697	PITPNM1	67266486	0.028351	0.095177	-2.1923
				ENSG00000110711	AIP	67254543	0.25487	0.520821	1.1386
				ENSG00000172663	TMEM134	67234283	0.227278	0.485549	-1.2073
				ENSG00000269913	CTC-1337H24.1	67227561	0.9469	0.99533	-0.0666
				ENSG00000175544	CABP4	67223288	0.419551	0.678246	-0.8071
				ENSG00000175514	GPR152	67219486	0.841637	0.99533	-0.1997
				ENSG00000213402	PTPRCAP	67204259	0.382663	0.642327	0.873

ENSG00000175634	RPS6KB2	67199401	0.082	0.226705	-1.7391
ENSG00000172531	PPP1CA	67177154	0.002583	0.011038	3.0134
ENSG00000172508	CARNS1	67187758	0.477084	0.700717	-0.711
ENSG00000175463	TBC1D10C	67174473	0.057775	0.169715	-1.8973
ENSG00000172613	RAD9A	67162528	0.613278	0.820949	-0.5052
ENSG00000175505	CLCF1	67136643	0.757778	0.937252	0.3084
ENSG00000110075	PPP6R3	68305494	0.179207	0.421137	1.3433
ENSG00000175482	POLD4	67121345	0.282643	0.53137	1.0745
ENSG00000172830	SSH3	67075498	0.121911	0.301571	-1.5467
ENSG00000172932	ANKRD13D	67062987	0.027426	0.095177	-2.2053
ENSG00000173020	ADRBK1	67043954	0.68254	0.86701	0.4091
ENSG00000260808	CTD-2007L18.5	68382273	0.893244	0.99533	0.1342
ENSG00000179038	AP001885.1	66964014	0.111925	0.292249	1.5897
ENSG00000173120	KDM2A	66955940	0.365359	0.635996	0.9053
ENSG00000132749	MTL5	68496970	0.997128	0.997128	-0.0035
ENSG00000110057	UNC93B1	67765513	0.000232	0.001092	-3.6809
ENSG00000110090	CPT1A	68566983	0.04056	0.127088	-2.0479
ENSG00000006534	ALDH3B1	67786396	2.86E-19	4.48E-18	-8.974
ENSG00000197345	MRPL21	68665023	1.60E-30	7.5E-29	11.4837
ENSG00000132740	IGHMBP2	68689690	2.43E-17	2.85E-16	-8.4711
ENSG00000162341	TPCN2	68837218	0.628812	0.820949	0.4835
ENSG00000259799	RP11-554A11.9	68925299	0.002932	0.011483	-2.9747

Supplementary Table 6 – Chromosome 6p21.32 risk locus for acute myeloid leukemia (AML) stratified by *NPM1* and *FLT3* mutation status. Table shows fixed-effects meta odds ratios for the risk locus at 6p21.32 (rs3916765) stratified by *NPM1* and *FLT3* mutation status. *NPM1* and *FLT3* mutation status was available for cases recruited to GWAS 2 and GWAS 4 only. For each GWAS, association tests were performed for cytogenetically normal AML cases assuming an additive genetic model, with nominally significant principal components included in the analysis as covariates. Association summary statistics were combined for variants common to GWAS 2 and GWAS 4, in fixed effects models using PLINK. All statistical tests were two-sided and no adjustments were made for multiple comparisons. N, number; OR, odds ratio; 95% CI, 95% confidence interval; Q, Cochran's Q statistic; I^2, heterogeneity index I^2.

AML risk variant	AML cytogenetics	Mutation status	Case, N / Control, N	GWAS, N	OR (95% CI)	*P* value	I^2	*P*Q
rs3916765	All AML	All cases	4018/10488	4	1.20 (1.09-1.32)	1.15e-04	37	0.187
		NPM1-negative	422/6205	2	1.28 (0.97-1.68)	0.08	0.00	0.62
		NPM1-positive	231/6205	2	1.96 (1.29-2.98)	1.7e-03	1.12	0.94
					Test for heterogeneity		63.88	0.10
		FLT3-negative	594/6205	2	1.26 (1.01-1.58)	0.04	0.00	0.85
		FLT3-positive	271/6205	2	1.52 (1.07-2.16)	0.02	81.37	0.02
					Test for heterogeneity		0.00	0.38
	Normal	All cases	1287/10488	4	1.72 (1.46-2.03)	1.51e-10	26	0.256
		NPM1-negative	205/6205	2	1.32 (0.91-1.92)	0.14	0.00	0.63
		NPM1-positive	206/6205	2	1.95 (1.26-3.01)	2.8e-03	0.00	0.96
					Test for heterogeneity		43.74	0.18
		FLT3-negative	328/6205	2	1.49 (1.10-2.02)	0.01	0.00	0.38
		FLT3-positive	200/6205	2	1.84 (1.20-2.81)	5.1e-03	0.00	0.52
					Test for heterogeneity		0.00	0.43

Supplementary Table 7- *cis*-eQTL analysis of rs10789158 in whole blood. *cis*-eQTL data for loci annotated to within 500Kb of rs10789158 derived from whole blood expression data collated by the eQTLGen Consortium (http://www.eqtlgen.org/cis-eqtls.html). SNP, single nucleotide polymorphism; QTL, expression quantitative trait loci; Chr, chromosome. [a]hg19 coordinates; [b]Unadjusted *P* value; [c]Benjamini-Hochberg corrected *P* value.

**SNP**	**SNP Position[a]**	**Assessed allele eQTL**	**Chr**	**Gene**	**Gene Symbol**	**Gene Position[a]**	**P value eQTL[b]**	**PBH eQTL[c]**	**Z score**
rs10789158	64867602	C	1	ENSG00000162433	AK4	65655530	0.0019144	0.012561175	3.1032
				ENSG00000226891	RP11-182I10.3	65453033	0.04860961	0.121411686	1.9721
				ENSG00000088035	ALG6	63868747	0.05518713	0.121411686	1.9175
				ENSG00000079739	PGM1	64092431	0.11328477	0.207688745	1.5838
				ENSG00000142856	ITGB3BP	63982916	0.45349565	0.623556519	0.7498
				ENSG00000162434	JAK1	65365549	0.67463156	0.742094716	0.4199
				ENSG00000185031	RP11-182I10.4	65451030	0.76265206	0.76265206	-0.3019
				ENSG00000158966	CACHD1	65047584	0.61468307	0.742094716	-0.5033
				ENSG00000203965	EFCAB7	64013703	0.31163369	0.489710084	-1.0117
				ENSG00000116675	DNAJC6	65797727	0.02633742	0.09657054	-2.221
				ENSG00000162437	RAVER2	65254846	0.00228385	0.012561175	-3.0505

Supplementary Table 8 - *cis*-eQTL analysis of rs17773014 in whole blood. *cis*-eQTL data for loci annotated to within 500Kb of rs17773014 derived from whole blood expression data collated by the eQTLGen Consortium (http://www.eqtlgen.org/cis-eqtls.html). SNP, single nucleotide polymorphism; QTL, expression quantitative trait loci; Chr, chromosome. [a]hg19 coordinates; [b]Unadjusted *P* value; [c]Benjamini-Hochberg corrected *P* value.

**rs17773014**	**134093813**	**G**	**7**	**ENSG00000205060**	**SLC35B4**	**133987943**	**0.02245394**	***0.06736182***	**2.2827**
				ENSG00000085662	AKR1B1	134135569	5.92E-24	5.32863E-23	-10.0931
				ENSG00000155530	LRGUK	133880697	0.84461017	0.950186441	-0.196
				ENSG00000172331	BPGM	134348062	0.30442046	0.524218755	-1.0269
				ENSG00000122786	CALD1	134542241	0.99888296	0.99888296	-0.0012
				ENSG00000131558	EXOC4	133344585	0.08572368	0.19287828	-1.7184
				ENSG00000146859	TMEM140	134841737	0.34947917	0.524218755	0.9357
				ENSG00000122783	C7orf49	134816331	0.40937159	0.526334901	-0.825
				ENSG00000105875	WDR91	134882453	0.01182917	0.053231265	-2.517

Supplementary Table 9 – Ethical approvals for studies contributing AML cases and/or controls to GWAS 1, GWAS 2, GWAS 3 and GWAS 4.

Cohort	Ethics Committee providing approval	Decision
GWAS 1 Cases		
UK MRC/NCRI AML clinical trials (N=529)	NHS York Research Ethics committee (REF 06/Q1108/92) and Newcastle University FMS Ethics Committee (REF BH36664 (7078)	Approved
Eurobank transplant study (N=70)	NHS North East Newcastle and North Tyneside 1 Research Ethics Committee	Approved
Leukaemia and Lymphoma Research adult acute Leukaemia study (N=222)	NHS York Research Ethics committee (REF 06/Q1108/92) and Newcastle University FMS Ethics Committee (REF BH36664 (7078)	Approved
Newcastle University Haematology Biobank (N=48)	NHS North East - Newcastle and North Tyneside 1 Research Ethics Committee (REF 17/NE/0361)	Approved
Institutional biobank of University of Chicago (N=250)	University of Chicago Medical Center Institutional Review Board	Approved
GWAS 1 Controls		
British 1958 Birth Cohort (N=2671)	NHS North West – Haydock Research Ethics Committee (REF 14/NW/1179)	Approved
GWAS 2 Cases		
Central England Haemato-Oncology and Oncology Research Bank (N=120)	NHS East Midlands - Derby Research Ethics Committee (REF 09/H0405/12+5)	Approved
King's College London Medical School (N=144)	NHS Wales Multicentre Research Ethics Committee (REF 08/MRE09/29)	Approved
Study Alliance Leukemia biobank at Dresden University (N=208)	Institutional Review Board of the Medical Faculty of Dresden (Medizinische Fakultat Dresden; IRB #EK98032010)	Approved
CALGB 9710 APL clinical trial (N=101)	University of Chicago Medical Center Institutional Review Board (REF CALGB 9710; NCT00003934)	Approved
Newcastle University Haematology Biobank (N=248)	NHS North East - Newcastle & North Tyneside Research Ethics Committee (REF 17/NE/0361)	Approved
Munich Leukemia Laboratory (N=53)	Bavarian Medical Association (Bayerische Landesärztekammer)(REF 5117)	Approved
Medical University of Graz (N=32)	Ethics Committee of the Medical University of Graz (REF 26-369 ex 13/14)	Approved
University Hospital Rigshospitalet Copenhagen (N=13)	The Capital Region of Denmark Ethic Committee (Historic samples - ethical approval not required)	Waived

Universita Cattolica Sacro Cuore Rome (N=12)	Institutional Review Board of the Policlinico Tor Vergata of Rome	Approved
GWAS 2 Controls		
UK Blood Service Control Group (N=2477)	NHS South East multicentre research ethics committee (REF 05/Q0106/74)(UK Blood Services Controls)	Approved
GWAS 3 Cases		
University of Heidelberg, Germany (N=82)	Medical Ethics Committee II of the Medical Faculty Mannheim of the Heidelberg University (REF 2013-509N-MA)	Approved
Centre Hospitalier Universitaire Nice, France (N=42)	Comité de Protection des Personnes Centre Hospitalier Universitaire de Nice (REF NCT01210274)	Approved
University of London UK (GSE20672)(N=40)	Data is in the public domain	Not applicable
Austrian Academy of Sciences (N=223)	Ethics Committee of the Medical University of Vienna	Approved
ALFA Clinical Trials at the University of Lille, France (N=278)	Institutional Review Borard of CHRU of Lille (CSTMT089)	Approved
Biobank La Fe at the Hospital Universitari i Politècnic La Fe, Spain (N=15)	Research Ethics Board of the Hospital Universitari i Politècnic La Fe, Spain (CEIB; Comité Ético de Investigación Biomédica).	Approved
German-Austria AML Study Group (GSE32462, N=189[70)]	Data is in the public domain	Not applicable
German-Austria AML Study Group (GSE34542, N=33[71])	Data is in the public domain	Not applicable
German-Austria AML Study Group (GSE46745, N=33)	Data is in the public domain	Not applicable
German-Austria AML Study Group (GSE46951, N=51)	Data is in the public domain	Not applicable
KORA study (N=5)	Ethics Committee of the Bavarian Medical Association and the Bavarian commissioner for data protection and privacy.	Approved
GWAS 3 Controls		
KORA study (N=1612)	Ethics Committee of the Bavarian Medical Association and the Bavarian commissioner for data protection and privacy.	Approved
GWAS 4 Cases		
Central England Haemato-Oncology and Oncology Research Bank (N=515)	NHS East Midlands - Derby Research Ethics Committee (REF 09/H0405/12+5)	Approved
Hematology Division at Semmelweis University, Budapest (N=202)	Ethical Committee of the Hungarian Medical Research Council (REF 45371-2/2016/EKU)	Approved

UK Biobank (N=260)	NHS North West Multi-centre Research Ethics Committee (MREC)(REF 16/NW/0274)	Approved
GWAS 4 Controls		
UK Biobank (N=3728)	NHS North West Multi-centre Research Ethics Committee (MREC)(REF 16/NW/0274)	Approved

Supplementary Table 10 – Primers used for *NPM1* and *FLT3* sequence analysis

NPM1 Fragment analysis primer sequences	
NPM1 Frag Forward	5’ TGT CTA TGA AGT GTT GTG GTT CC 3’
NPM1 Frag Reverse	5’ VIC-AAA AAG GAC AGC CAG ATA TCA A 3’
NPM1 Sequencing primers	
NPM1 Exon 12 Forward	5’ TGT CTA TGA AGT GTT GTG GTT CC 3’
NPM1 Exon 12 Reverse	5’ TTT GGA CAA CAC ATT CTT 3’
FLT3 Sequencing primers	
FLT3 Forward	5’ 6-FAM-GCA ATT TAG GTA TGA AAG CCA GC 3’
FLT3 Reverse	5’ CTT TCA GCA TTT TGA CGG CAA CC 3’
