# Supplementary material for: Genome-wide association study identifies susceptibility loci for acute myeloid leukemia
Source: Nat Commun. 2021 Oct 29;12:6233. doi: 10.1038/s41467-021-26551-x (PMC8556284; doi:10.1038/s41467-021-26551-x)
Supplement: Supplementary file 3 — Reporting summary [file 41467_2021_26551_MOESM3_ESM.pdf]

nature research

Corresponding author(s): Friedrich Stolzel, Kenan Onel, James Allan

Last updated by author(s): Sep 28, 2021

Reporting Summary

Nature Research wishes to improve the reproducibility of the work that we publish. This form provides structure for consistency and transparency in reporting. For further information on Nature Research policies, see Authors & Referees and the Editorial Policy Checklist .

Statistics

For all statistical analyses, confirm that the following items are present in the figure legend, table legend, main text, or Methods section.

n/a	Confirmed	
[ ]	[x]	The exact sample size (*n*) for each experimental group/condition, given as a discrete number and unit of measurement
[x]	[ ]	A statement on whether measurements were taken from distinct samples or whether the same sample was measured repeatedly
[ ]	[x]	The statistical test(s) used AND whether they are one- or two-sided<br>*Only common tests should be described solely by name; describe more complex techniques in the Methods section.*
[ ]	[x]	A description of all covariates tested
[ ]	[x]	A description of any assumptions or corrections, such as tests of normality and adjustment for multiple comparisons
[ ]	[x]	A full description of the statistical parameters including central tendency (e.g. means) or other basic estimates (e.g. regression coefficient) AND variation (e.g. standard deviation) or associated estimates of uncertainty (e.g. confidence intervals)
[ ]	[x]	For null hypothesis testing, the test statistic (e.g. *F*, *t*, *r*) with confidence intervals, effect sizes, degrees of freedom and *P* value noted<br>*Give P values as exact values whenever suitable.*
[x]	[ ]	For Bayesian analysis, information on the choice of priors and Markov chain Monte Carlo settings
[x]	[ ]	For hierarchical and complex designs, identification of the appropriate level for tests and full reporting of outcomes
[x]	[ ]	Estimates of effect sizes (e.g. Cohen's *d*, Pearson's *r*), indicating how they were calculated

Our web collection on statistics for biologists contains articles on many of the points above.

Software and code

Policy information about availability of computer code

Data collection	No software was used.
Data analysis	Genotyping and genome-wide quality-control procedures<br>Genotype calling was performed using Illumina GenomeStudio software v2.0 or Affymetrix Genotyping Console software v4.2.0.26. Data handling and analysis was performed using R v3.5.1, PLINK v1.9b4.4 and SNPTEST v2.5.2. Rigorous SNP and sample quality control metrics were applied to all four GWAS. Specifically, we excluded SNPs with extreme departure from Hardy-Weinberg equilibrium (HWE; P < 10-3 in either cases or controls) and with a low call rate (< 95%). We also excluded SNPs that showed significant differences (P < 10-3) between genotype batches and with significant differences (P < 0.05) in missingness between cases and controls. Individual samples with a call rate of < 95% or with extreme heterozygosity rates (+/- 3 standard deviation from the mean) were also excluded from each GWAS. Individuals were removed such that there were no two individuals with estimated relatedness pihat > 0.1875, both within and across GWAS. The individual with the higher call rate was retained unless relatedness was identified between a case and a control, where the case was preferentially retained. Ancestry was assessed using principal component analysis and super-populations from the 1000 genomes project as a reference, with individuals of non-European ancestry excluded based on the first two principal components. In order to minimise any impact of population stratification among the European population we excluded outlying cases and controls identified using principal components 1 and 2 for each GWAS.<br><br>Imputation, genome-wide association testing and meta-analysis<br>Genome-wide imputation for each GWAS was performed using the Michigan Imputation Server (https://imputationserver.sph.umich.edu/index.html) and the Haplotype Reference Consortium reference haplotype panel (http://www.haplotype-reference-consortium.org/) following pre-phasing using ShapeIT (v2.r790). All variants with an imputation info score < 0.6 or a minor allele frequency of < 0.01 were excluded from subsequent analysis.<br><br>For each GWAS, association tests were performed for all cases and cytogenetically normal AML assuming an additive genetic model, with nominally significant principal components included in the analysis as covariates. Association summary statistics were combined for variants common to GWAS 1, GWAS 2 and GWAS 3, and then for variants common to all four GWAS, in fixed effects models using PLINK

v1.9b4.4. Cochran's Q statistic was used to test for heterogeneity and the I2 statistic was used to quantify variation due to heterogeneity.

The Bayesian False Discovery Probability was calculated using a prior probability of association of 0.0001 and a plausible OR of 1.39.

Technical validation of AML susceptibility variants
All four AML risk variants reported here were either directly genotyped or imputed to high quality. Specifically, rs4930561 was directly genotyped in GWAS 1 and GWAS 2 and imputed in GWAS 3 and GWAS 4 (info score 0.974 - 0.988); rs3916765 was genotyped in GWAS 4 and imputed in GWAS 1, GWAS 2 and GWAS 3 (info score 0.901-0.995); rs10789158 was imputed in all 4 GWAS studies (info score 0.946-0.9775); and rs17773014 was directly genotyped in GWAS 3 and GWAS 4 and imputed in GWAS 1 and GWAS 2 (info score 0.985-0.993). Fidelity of array genotyping and imputed dosages was confirmed using Sanger sequencing in a subset of AML samples (including samples genotyped on both Illumina and Affymetrix platforms) for each sentinel variant with perfect or very high concordance for all four variants.

The majority of AML cases were genotyped using DNA extracted from cell/tissue samples (blood and bone marrow) taken during AML remission. A minority of AML cases were genotyped using DNA extracted from tissue samples that include leukemic AML cells. As such, we employed a stringent HWE cut-off in order to eliminate SNPs potentially affected by somatic copy number alterations. Furthermore, we also used Nexus Copy Number v10 (BioDiscovery, California) to interrogate B allele frequency and Log R ratio values at loci associated with AML following genotyping of DNA extracted from leukemic AML cells. For rs4930561 (chromosome 11q13.2) we interrogated data from 352 AML cases using samples with high somatic cell content and found one case with a large deletion capturing the KMT5B locus. We also identified 12 cases with evidence of trisomy 11 or large gains affecting chromosome 11, consistent with reports of trisomy 11 in approximately 1% of AML cases. For rs10789158 (chromosome 1p31.3) we identified 1 case with evidence of copy number gain. The susceptibility locus at chromosome 1 does not fall within a region reported to be recurrently somatically deleted or amplified in AML. The association signals at 6p21.32 (rs3916765) and 7q33 (rs17773014) were specific to cytogenetically normal AML and evidence of somatic copy number alterations were visible in 0 and 3 cases, respectively (based on Nexus Copy Number analysis of 127 cytogenetically normal AML cases). Specifically, there were three cases with evidence of deletions affecting the chromosome 7 risk locus that were not visible cytogenetically. Furthermore, there was no evidence of copy neutral loss of heterozygosity (>2 Mb) at any of the four AML susceptibility loci reported here. Taken together, these data limit the possibility of differential genotyping in cases and controls due to somatically acquired allelic imbalance.

HLA imputation, expression quantitative trait loci (eQTL) analysis and functional annotation
Imputation of classical HLA alleles was performed using the SNP2HLA v1.0.3 tool using 5225 Europeans from the Type I Diabetes Genetics Consortium as a reference panel. To examine the relationship between SNP genotype and gene expression and identify cis expression quantitative trait loci (eQTLs) we made use of data from the eQTLGen Consortium (http://www.eqtlgen.org/cis-eqtls.html) for whole blood. Benjamini-Hochberg (BH)-adjusted P values were estimated for each gene annotated to within 1Mb of the sentinel SNP at each AML association signal. Regions with AML susceptibility variants were annotated for putative functional motifs using data from the ENCODE project.

Relationship between SNP genotype and patient survival
The relationship between AML risk variants and survival was evaluated in a total of 767 AML patients (excluding acute promyelocytic leukemia) from the UK, Germany and Hungary. Briefly, patients were treated with conventional intensive AML therapy including ara-C, daunorubicin and best supportive care. A subset of high-risk patients in the German cohort were treated with stem cell transplantation. Overall survival was defined as the time from diagnosis to the date of last follow-up or death from any cause. Data on relapse-free survival was available on 358 AML patients, which was defined as the time from date of first remission to the date of last follow-up in remission or date of AML relapse. Cox regression analysis was used to estimate allele specific hazard ratios and 95% confidence intervals for each study in analyses that included all AML cases (N=767) and cytogenetically normal AML (N=358).

For manuscripts utilizing custom algorithms or software that are central to the research but not yet described in published literature, software must be made available to editors/reviewers. We strongly encourage code deposition in a community repository (e.g. GitHub). See the Nature Research guidelines for submitting code & software for further information.

Data

Policy information about availability of data

All manuscripts must include a data availability statement. This statement should provide the following information, where applicable:

- Accession codes, unique identifiers, or web links for publicly available datasets
- A list of figures that have associated raw data
- A description of any restrictions on data availability

Genome-wide association summary statistics (Lin_AML_metaassoc.txt) are available for download from https://doi.org/10.25405/data.ncl.16558116.v1. AML case and control genotyping data from the UK Biobank can be obtained via application through https://www.ukbiobank.ac.uk/. Genotyping data on 2699 individuals recruited to the 1958 British Birth Cohort (Hap1.2M-Duo Custom array data) and 2501 individuals from the UK Blood Service are available from the Wellcome Trust Case Control Consortium 2 [https://www.wtccc.org.uk/;WTCCC2:EGAD00000000022,%20EGAD00000000024]. Case and control genotyping data from 1615 individuals recruited to the KORA study can be obtained via application at https://www.helmholtz-muenchen.de/en/kora/. Other genotyping data supporting the findings of this study can be found as deposited in NCBI Gene Expression Omnibus under accession numbers GSE20672 [https://www.ncbi.nlm.nih.gov/geo/query/acc.cgi?acc=GSE20672], GSE32462 [https://www.ncbi.nlm.nih.gov/geo/query/acc.cgi?acc=GSE32462], GSE34542 [https://www.ncbi.nlm.nih.gov/geo/query/acc.cgi?acc=GSE34542], GSE46745 [https://www.ncbi.nlm.nih.gov/geo/query/acc.cgi?acc=GSE46745] and GSE46951 [https://www.ncbi.nlm.nih.gov/geo/query/acc.cgi?acc=GSE46951]. eQTL data is available from the eQTLGen consortium via http://www.eqtlgen.org/cis-eqtls.html. ENCODE data is available from https://www.encodeproject.org/biosamples/ENCBS718AAA/ for H1 human embryonic stem cells (H1hesc) and from https://www.encodeproject.org/biosamples/ENCBS109ENC/ for K562 myeloid leukemia cells. URLs: Michigan Imputation Server, https://imputationserver.sph.umich.edu/index.html#!; Haplotype Reference Consortium, http://www.haplotype-reference-consortium.org/; eQTLGen Consortium, http://www.eqtlgen.org/cis-eqtls.html; 1000 Genomes Project, https://www.internationalgenome.org/; PLINK, https://www.cog-genomics.org/plink2/; SNPTEST2, https://www.well.ox.ac.uk/~gav/snptest/; Phenoscanner, http://www.phenoscanner.medschl.cam.ac.uk/ 76; UK Biobank, https://www.ukbiobank.ac.uk/; Central England Haemato-Oncology and Oncology Research Bank, https://

bwc.nhs.uk/central-england-haemato-oncology-and-oncology-research-bioba/; Newcastle University Biobank, https://www.ncl.ac.uk/biobanks/; WTCCC, https://www.wtccc.org.uk/; ENCODE, https://www.encodeproject.org/; Study Alliance Leukemia, https://www.sal-aml.org/ueber-uns/einfuehrung-ueberblick

Field-specific reporting

Please select the one below that is the best fit for your research. If you are not sure, read the appropriate sections before making your selection.

- [x] Life sciences
- [ ] Behavioural & social sciences
- [ ] Ecological, evolutionary & environmental sciences

For a reference copy of the document with all sections, see nature.com/documents/nr-reporting-summary-flat.pdf

Life sciences study design

All studies must disclose on these points even when the disclosure is negative.

Sample size	Observational study, so the results are based on all available data from acute myeloid leukemia patients recruited to participating centres. This study includes data on 4018 new cases.
Data exclusions	Data were quality controlled using standard protocols and criteria internationally accepted for genome-wide association studies. Specifically, for each GWAS we excluded SNP markers with departure from Hardy-Weinberg equilibrium (HWE; P ≤ 10-3), a call rate < 95% or with significant differences in minor allele frequency (P ≤ 10-3) between genotype batches. Samples were excluded if the call rate was < 95%, heterozygosity exceeded 3 standard deviations from the overall mean heterozygosity or were identified as non-European based on principal components analysis using 1000 genome data as a reference. Samples were also removed such that there were no two individuals with estimated relatedness pihat ≥ 0.1875, with retention of the sample with the higher call rate.
Replication	Observational study, so the results based on all available data. The reported AML risk variants replicate in four independent genome-wide association studies (GWAS) and achieve genome-wide statistical significance in meta-analysis (P < 5 x 10^-8), consistent with the international standard for reporting risk variants in GWAS.
Randomization	Observational study, so randomisation not relevant. Sample recruitment based on a diagnosis of acute myeloid leukemia.
Blinding	Observational study, so blinding not relevant. Sample recruitment based on a diagnosis of acute myeloid leukemia.

Reporting for specific materials, systems and methods

We require information from authors about some types of materials, experimental systems and methods used in many studies. Here, indicate whether each material, system or method listed is relevant to your study. If you are not sure if a list item applies to your research, read the appropriate section before selecting a response.

Materials & experimental systems

n/a	Involved in the study	
x		Antibodies
x		Eukaryotic cell lines
x		Palaeontology
x		Animals and other organisms
	x	Human research participants
x		Clinical data

Methods

n/a	Involved in the study	
x		ChIP-seq
x		Flow cytometry
x		MRI-based neuroimaging

Human research participants

Policy information about studies involving human research participants

Population characteristics	All patients had typical acute myeloid leukemia were diagnosed in accordance with World Health Organisation guidelines contemporaneous to the time of diagnosis. Patient characteristics are described in the methods section and supplementary table 1 of the manuscript.
Recruitment	Samples and data from acute myeloid leukemia patients were accessed via biobanks and clinical centres across Europe and North America. The results are based on chronic lymphocytic leukemia patients daignosed at participating centres/studies for whom a DNA sample was available. As such, there are no biases in recruitment (or other aspect) that could affect the study.
Ethics oversight	Collection of patient samples and associated clinico-pathological information was undertaken with written informed consent. All studies were conducted in accordance with the Declaration of Helsinki and received local institutional review board or national research ethics approval, as appropriate. Specifically, this research has been conducted using the UK Biobank Resource (Application #16583, James Allan). MRC/NCRI AML 11 trial, AML 12 trial and the UK Leukaemia Research Fund (LLR) population-based case-control study of adult acute leukemia received multicenter research ethics committee approval, as previously described54, 55, 75. Research ethics committee approval was given to the Newcastle Haematology Biobank (07/H0906/109+5) and the AML genome-wide association study in the UK (06/q1108/92, BH136664 (7078)). AML cases and controls for Samples from the Hungarian AML patients were obtained during the standard diagnostic workup at the Hematology Divisions of the 1st and 3rd Department of Internal Medicine, Semmelweis University, Budapest, following ethical approval from the Local Ethical Committee (TUKEB-1552012) and the Hungarian Medical Research Council (1483-2/2017/EKU). Saliva and fibroblast samples from Austrian AML patients were collected at the Division of Hematology, Medical University of Graz, Graz, Austria, and processed as described76. The diagnosis of AML was made in accordance with World Health Organisation guidelines.

Note that full information on the approval of the study protocol must also be provided in the manuscript.
